# Rare Earth Doped Ceria: The Complex Connection Between Structure and Properties

**DOI:** 10.3389/fchem.2018.00526

**Published:** 2018-10-31

**Authors:** Mauro Coduri, Stefano Checchia, Mariangela Longhi, Davide Ceresoli, Marco Scavini

**Affiliations:** ^1^ESRF - The European Synchrotron, Grenoble, France; ^2^Dipartimento di Chimica, Università degli Studi di Milano, Milan, Italy; ^3^Istituto di Scienze e Tecnologie Molecolari, CNR, Milan, Italy

**Keywords:** rare earths doped ceria, energy, defects chemistry, structure, diffraction, microscopy, spectroscopy, theoretical calculations

## Abstract

The need for high efficiency energy production, conversion, storage and transport is serving as a robust guide for the development of new materials. Materials with physical-chemical properties matching specific functions in devices are produced by suitably tuning the crystallographic- defect- and micro-structure of the involved phases. In this review, we discuss the case of Rare Earth doped Ceria. Due to their high oxygen diffusion coefficient at temperatures higher than ~500°C, they are very promising materials for several applications such as electrolytes for Solid Oxide Fuel and Electrolytic Cells (SOFC and SOEC, respectively). Defects are integral part of the conduction process, hence of the final application. As the fluorite structure of ceria is capable of accommodating a high concentration of lattice defects, the characterization and comprehension of such complex and highly defective materials involve expertise spanning from computational chemistry, physical chemistry, catalysis, electrochemistry, microscopy, spectroscopy, and crystallography. Results coming from different experimental and computational techniques will be reviewed, showing that structure determination (at different scale length) plays a pivotal role bridging theoretical calculation and physical properties of these complex materials.

## Introduction

Pure and doped cerium oxides have high catalytic, oxygen exchange and charge transport performances. The pure compound easily exchanges oxygen with the atmosphere and undergoes oxidation-reduction cycles, based on the Ce^3+^/Ce^4+^ redox couple, making CeO_2_ useful for many catalytic processes especially in nanostructured form, to maximize the surface to bulk ratio. On the other hand, doping with lower valent cations (e.g., trivalent rare earth cations, RE^3+^) depresses Ce^3+^ concentration and introduces a huge amount of oxygen vacancies (V_O_s) so increasing the O diffusion coefficient, paving the way to high ionic conductivity at reasonable low *T*-values (500–700°C). Hence, RE-doped CeO_2_ solid solutions are candidates for applications as electrodes and/or electrolytes in Solid Oxide Fuel Cells (SOFC) and Electrolysis Cells (SOEC).

Nevertheless, doping introduces a large amount of defects which form complex and hierarchical architectures depending on dopant nature and amount, crystallite size, and even synthetic path. Defects architectures deeply affect the structure at different length scales with not obvious consequences on physical properties.

The combination of technological interest and tricky scientific problems attracted the attention of the scientific community in the last decades like flies to honey resulting in hundreds of research and review papers covering a wide part of material science spectrum. In fact, the characterization and comprehension of such complex and highly defective materials involve expertise spanning from computational chemistry to physical chemistry, catalysis, electrochemistry, spectroscopy, microscopy and crystallography making a hard task to build a *summa* of existing literature.

We do not pretend to climb such a high mountain. This review addresses the close relationship among defect chemistry, structure, and physical properties. Some recent results from various experimental and computational techniques will be reviewed, showing that structure determination (at different scale length) plays a pivotal role bridging theoretical calculations and physical properties.

After a brief introduction on technological applications, we will introduce the defect chemistry of pure and doped cerium oxide. Then structural, spectroscopic and computational tools adopted to investigate them are reviewed and discussed.

## Technological applications of CeO_2_-based materials

Ceria is one of the most studied mixed ionic and electronic conducting materials and benefits of outstanding redox properties associated to the easy interconversion between Ce(III) and Ce(IV) (Trovarelli, 1996). Its applications span from three-way catalyst in automotive industry to electrolyte in Solid Oxide Fuel Cells (Montini et al., 2016).

SOFCs at intermediate (500° < T < 700°C) and high temperature (T>800°C) have high energy conversion efficiency and high compatibility with many fuels without suffering from CO poisoning. They are promising devices for innovative energy applications where ceria derivatives can be used in different ways, as a catalyst in both cathodes and anodes, as protective layer on cathodes to limit aggressive action of Y_2_O_3_ stabilized ZrO_2_ (ZYO) electrolyte, and as electrolyte (Montini et al., 2016).

**Figure 1:**
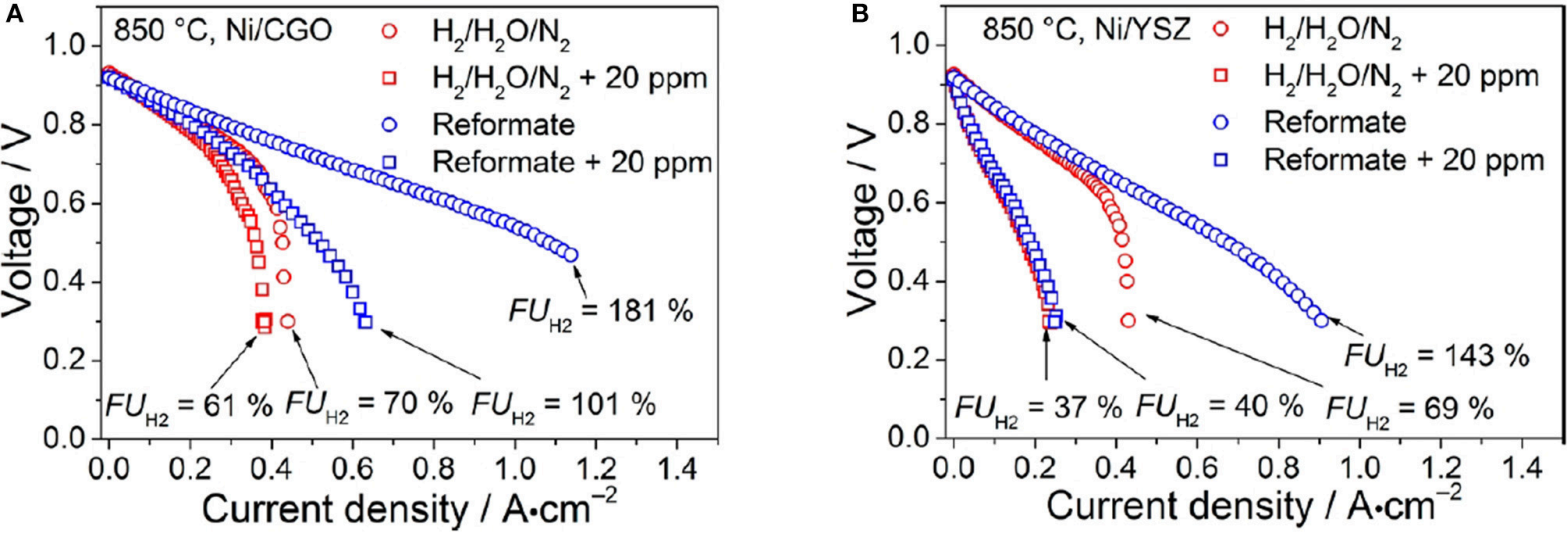
i–V curves of the **(A)** Ni/CGO- and **(B)** Ni/YSZ-based cells. Experiments were conducted at 850°C. The reformate consisted of gas mixture IV (blue) containing 7% H_2_, 7% H_2_O, 20% CO, 20% CO_2_, and 46% N_2_, and the reference mixture V (red) consisted of 7% H_2_, 7% H_2_O, 86% N_2_. i–V curves were recorded with (squares) and without (circles) the addition of 20 ppm of H_2_S. Reprinted with permissions from Riegraf et al. (2017). © 2017 American Chemical Society.

Ceria is a very interesting anodic material thanks to its capability of oxidizing carbon containing fuels (Park et al., 1999; McIntosh and Gorte, 2004) while still showing an extended electrochemically active area. Although undoped CeO_2_ is not a good ionic conductor, doping with lower valent oxides, like e.g., Samaria, induces the formation of V_O_s thus increasing oxygen ion conductivity thanks to a vacancy jump mechanism (Koettgen et al., 2018). Performance can be enhanced by improving the ionic conductivity of the anode (Zhu and Deevi, 2003). Similar results have been obtained for gadolinium doped ceria (CGO) (Nakamura et al., 2008; DeCaluwe et al., 2010; Chueh et al., 2011; Papaefthimiou et al., 2013; Feng et al., 2014), which is characterized by both high surface electroactivity toward H_2_ oxidation and mixed ionic/electronic conductivity at high temperatures (Nakamura et al., 2008; DeCaluwe et al., 2010; Chueh et al., 2011; Papaefthimiou et al., 2013; Feng et al., 2014; Riegraf et al., 2017). Anode tolerance to sulfur is an important property since sulfur, contained in SOFC fuel as natural gas and bio gas, is a detrimental poison for the cell efficiency (Riegraf et al., 2017). To overcome this problem and increase sulfur tolerance, Cu and Ni were added on the anode surface with promising results (He et al., 2005; Riegraf et al., 2017). The use of a Cu-CGO in H_2_-feeded SOFC maintains fuel cell performance in the presence of sulfur-based impurity levels up to 445 ppm (He et al., 2005). As to Ni-CGO anodes for CO conversion tolerance has been demonstrated at H_2_S concentrations up to 20 ppm (see Figure 1) and the sulfur poisoning behavior was reversible for the investigated short exposure times (Riegraf et al., 2017). Also, infiltration of CGO nanoparticles into porous Ni-CGO-based SOFC reduces sulfur poisoning and is beneficial to stabilizing the performances of SOFCs: infiltrated SOFCs show stable performance with sulfur contaminated fuel for over 290 h, while unmodified SOFCs become inoperative after 60 h (Hays et al., 2018).

Anodes based on Ni-Samaria-doped ceria (Ni-CSO) show better long-term durability and performance in SOFCs fueled with humidified methane than Ni-ZYO. This improvement was attributed to higher catalytic activity and electronic conductivity of Ni-CSO (Lee et al., 2013).

Another important parameter affecting SOFC performances is the morphology of the catalyst used as anode. As reported in Montini et al. (2016) Pd@CeO_2_ core–shell systems have good activity as anode in a SOFC fed by hydrogen and methane. The core–shell structure provides an extra stabilization enhancing high temperature performance (Adijanto et al., 2013).

Ceria can be also used as a protective layer on cathodes of SOFC to limit the aggressive action of ZYO electrolytes (Montini et al., 2016). Cathode in high-performance SOFCs are usually based on La_1−x_Sr_x_Co_1−y_Fe_y_O_3−δ_. When they are applied on ZYO an interlayer of SrZrO_3_ is formed, which modifies the resistance toward worse performance. This undesired reaction can be limited by introducing a layer of CGO between lanthanum-modified oxide and ZYO (Szász et al., 2017). This interlayer can interdiffuse in ZYO, improving performances, but it is strongly dependent on the CGO sintering temperature (Szász et al., 2017).

Due to their peculiar ionic conductivity, ceria and ceria-derivatives are extensively studied as solid electrolytes in SOFC for intermediate and low temperatures (Inaba and Tagawa, 1996). A high oxygen ion conductivity in the electrolyte is necessary to obtain good performance, and, among challenging materials, the very promising ones are rare-earth (RE) doped ceria Ce_1−x_RE_x_O_2−x/2_ (Mogensen et al., 2000). As observed above, an increase in ionic conductivity is obtained by increasing V_O_s through doping ceria. Gadolinium, samarium and yttrium are typical dopants (Steele, 2000; Montini et al., 2016). Bulk ionic conductivity of rare earths doped ceria will be the main subject of section Defect Chemistry and Transport Properties.

Crystal size is another important parameter since for polycrystalline samples bulk and grain boundary domains affect conductivity differently. In the bulk, oxygen ions jump through the regular lattice, but at grain boundaries they do along or across dislocations and in space charge zones. For small dopant fractions, grain boundary conductivity is low thus limiting the total conductivity. Instead, for large dopant concentration, the conductivity is defined by the low bulk conductivity. Nanostructuring causes a conductivity enhancement due to a larger contribution of grain-boundary conductivity in comparison with traditional polycrystalline solids (Koettgen et al., 2018) and also a better electrode activity (Tuller, 2000; Guo et al., 2002). Macroscopic charge transport through nanometric materials can be attained by means of high-pressure spark plasma sintering processes that form dense samples (>99%) without growing nanoparticles (Anselmi-Tamburini et al., 2006).

All these aspects are also important when cerium based materials are applied in SOECs, where they play the same role as in SOFC. In these devices hydrogen, carbon monoxide or syngas can be obtained by high temperature electrolysis of CO_2_ and H_2_O (Duboviks et al., 2014). However, there are drawbacks in SOECs based on zirconia-derived electrolyte that prevented their commercialization so far. The most important is the deposition of carbon at the electrode-electrolyte interface, demonstrated by Raman spectroscopy (Duboviks et al., 2014). Carbon at the interface reduces the number of active sites and, in the worst case, delaminate the interface (Navasa et al., 2018). This is especially relevant when the co-electrolysis of CO_2_ and H_2_O is performed at high current densities. The presence of an interlayer of ceria derivatives between Ni-based electrode and Ni-ZYO electrolyte improve performances (Navasa et al., 2018), owing to different carbon deposition characteristics of ceria and Ni-ZYO (Duboviks et al., 2015; Li et al., 2015; Hartvigsen et al., 2017).

## Defect chemistry and transport properties

The outstanding properties described above are closely related to the high oxygen diffusion coefficient and to the capability of pure and doped ceria of exchanging oxygen with the atmosphere. Being oxygen ions charged particles, this implies also high ionic conductivity and fast redox reactions. In this section we will present and briefly discuss the charge transport properties, focusing on bulk conductivity of polycrystalline samples. Readers interested in surface phenomena are referred to two recent reviews focused on ceria catalytic properties (Montini et al., 2016; Trovarelli and Llorca, 2017).

Firstly, we will introduce defect equilibria, limiting the analysis to zero-dimensional species, thus excluding one- and two-dimensional structures such as dislocations and antiphase boundaries (APB). Then, we will present conductivity experiments pointing out the open problems raised by the complex trends of conductivity σ as a function of defect nature and concentration.

### Defect chemistry

Starting from pure ceria, let's consider first the formation of anti-Frenkel (AF) defects: an oxygen ion jumps to an interstitial site, leaving an V_O_ at the O site (Mamontov and Egami, 2000). Following the Kröger-Vink notation (Kröger, 1977), the defect equation is:

(1) $$\begin{array}{l} O_{O}\vec{\leftarrow }V_{O}^{••}+O_{i}\prime \prime \end{array}$$

AF defects are supposed to be involved in the oxygen storage capacity of these materials, especially in nanocrystalline form (Mamontov et al., 2000).

V_O_s can be also formed by the interaction of CeO_2−δ_ with the atmosphere, following the equilibrium:

(2) $$\begin{array}{l} O_{O}\vec{\leftarrow }V_{O}^{••}+2e^{\prime }+\frac{1}{2}O_{2}\;\left(g\right) \end{array}$$

Oxygen deficiency δ values vs. temperature and oxygen partial pressure *p*O_2_ from different authors were reviewed by Mogensen et al. (2000).

For each V_O_, two electrons are injected in the conduction band (Tuller and Nowick, 1979). The pertinent equilibrium constant is:

(3) $$\begin{array}{l} K_{n}=\left[V_{O}^{••}\right]{\left[e^{\prime }\right]}^{2}pO_{2}^{1/2} \end{array}$$

where *p*O_2_ is the oxygen partial pressure. Electrons produced in equilibrium (2) form adiabatic small polarons and electronic conduction is achieved via an activated diffusive hopping mechanism involving Ce^4+^ to Ce^3+^ reduction (Tuller and Nowick, 1977; Chiang et al., 1996; Oliva et al., 2004; Farra et al., 2013). Electronic conductivity σ_e_ can be expressed as:

(4) $$\begin{array}{l} \sigma_{e}=n_{e}q\frac{\mu_{0}}{T}exp\left(-\frac{E_{H}}{kT}\right) \end{array}$$

In Equation (4), n_e_ and q are the electron concentration in the conduction band and electric charge, respectively, μ_0_ is the *T*-independent part of the electron mobility term, E_H_ is the hopping activation energy of the polaron and k is the Boltzmann's constant.

Considering the electro-neutrality condition $2\left[V_{O}^{••}\right]=\left[e^{\prime }\right]$, electron concentration in the conduction band and oxygen partial pressure are related by:

(5) $$\begin{array}{l} n_{e}=\left[e^{\prime }\right]=\sqrt[{3}]{2K_{n}}pO_{2}^{-1/6} \end{array}$$

Doubly ionized V_O_s are dominant at low δ, while defects interactions and single ionized V_O_s become important at larger δ (Tuller and Nowick, 1979), bringing to different equilibria (Mogensen et al., 2000). In any case, an oxygen partial pressure dependent conductivity is an efficient way to detect electronic contribution to charge transport in non-stoichiometric oxides (Scavini et al., 1994).

Upon doping ceria with trivalent RE ions, RE^3+^ substitute Ce^4+^ and V_O_s are introduced in the structure for charge compensation. Doping occurs during the synthesis process and the reaction can be described using the following equation:

(6) $$\begin{array}{l} \left(1-x\right)CeO_{2}+x/2{RE}_{2}O_{3}\;\vec{\leftarrow }\left(1-x\right){Ce}_{Ce}+x{RE}_{Ce}\prime \\ \;+\left(2-x/2\right)O_{O}+x/2V_{O}^{••} \end{array}$$

Equation (6) assumes trivalent cations only. If not, V_O_ concentration differs from *x*/2.

Ceria doped materials display high oxygen mobility via a vacancy diffusion mechanism and, as a consequence, high ionic conductivity σ_i_. σ_i_ can be expressed as Tuller and Nowick (1975):

(7) $$\begin{array}{l} \sigma_{i}=n_{i}\frac{\nu a^{2}q^{2}}{4kT}exp\left(\frac{{\Delta S}_{i}}{k}\right)exp\left(-\frac{E_{i}}{kT}\right)=\frac{\sigma_{0,i}}{T}exp\left(-\frac{E_{i}}{kT}\right) \end{array}$$

where $n_{i}=\left[V_{O}^{••}\right]$, is proportional to the probability to find a vacant site around the jumping oxygen ion, *E*_i_ and Δ*S*_i_ are the activation energy (Enthalpy) and Entropy for oxygen diffusion, respectively, *q* is the charge (=2e), k is the Boltzmann constant, υ is a frequency factor and *a*/2 (i.e., half of the cell parameter) is the jump distance for an V_O_, along the <100> crystallographic direction (Mogensen et al., 2000; Koettgen et al., 2018). According to equilibrium (6), RE doping introduces additional V_O_s; also, the presence of the charged *RE*′_Ce_ defects changes the electro-neutrality condition into $2[V_{O}^{••}]=\left[RE_{Ce}\prime \right]+\left[e\prime \right]$ and, as a consequence, the dependence *of n*_*e*_ vs. *p*O_2_ described in Equation (5) is turned into $n_{e}\propto pO_{2}^{-\frac{1}{4}}$ for $\left[RE_{Ce}\prime \right]\gg \left[e\prime \right]$ (Tuller and Nowick, 1975). Finally, for large *x* values, equilibrium (2) is pushed toward its left side and the concentration of conducting electrons is negligible but at very low oxygen partial pressures (see below).

### Transport properties

Electrochemical Impedance Spectroscopy (EIS) is usually adopted to measure conductivity in materials in which the ionic conduction is prevalent on the electronic one (Sacco, 2017). A small sinusoidal voltage V = V_0_sin(ωt) is applied and the response current I = I_0_sin(ωt+α) is measured at the same frequency. As a consequence of the (possible) phase shift α, the impedance Z calculated through Ohm's law is a complex number (Z = Z′ + iZ″ = V/I). A wide range of frequencies ω are sampled and Z data are typically plotted using the Nyquist representation. Data are then fitted against an equivalent circuit that is a combination of resistive R and capacitive C terms considering the transport across the bulk (R_b_ and C_b_) and the grain boundary (R_gb_ and C_gb_) of a polycrystal. Further elements are usually added for the electrode-electrolyte surface contribution and/or electronic transport in mixed ionic/electronic conductors.

**Figure 2:**
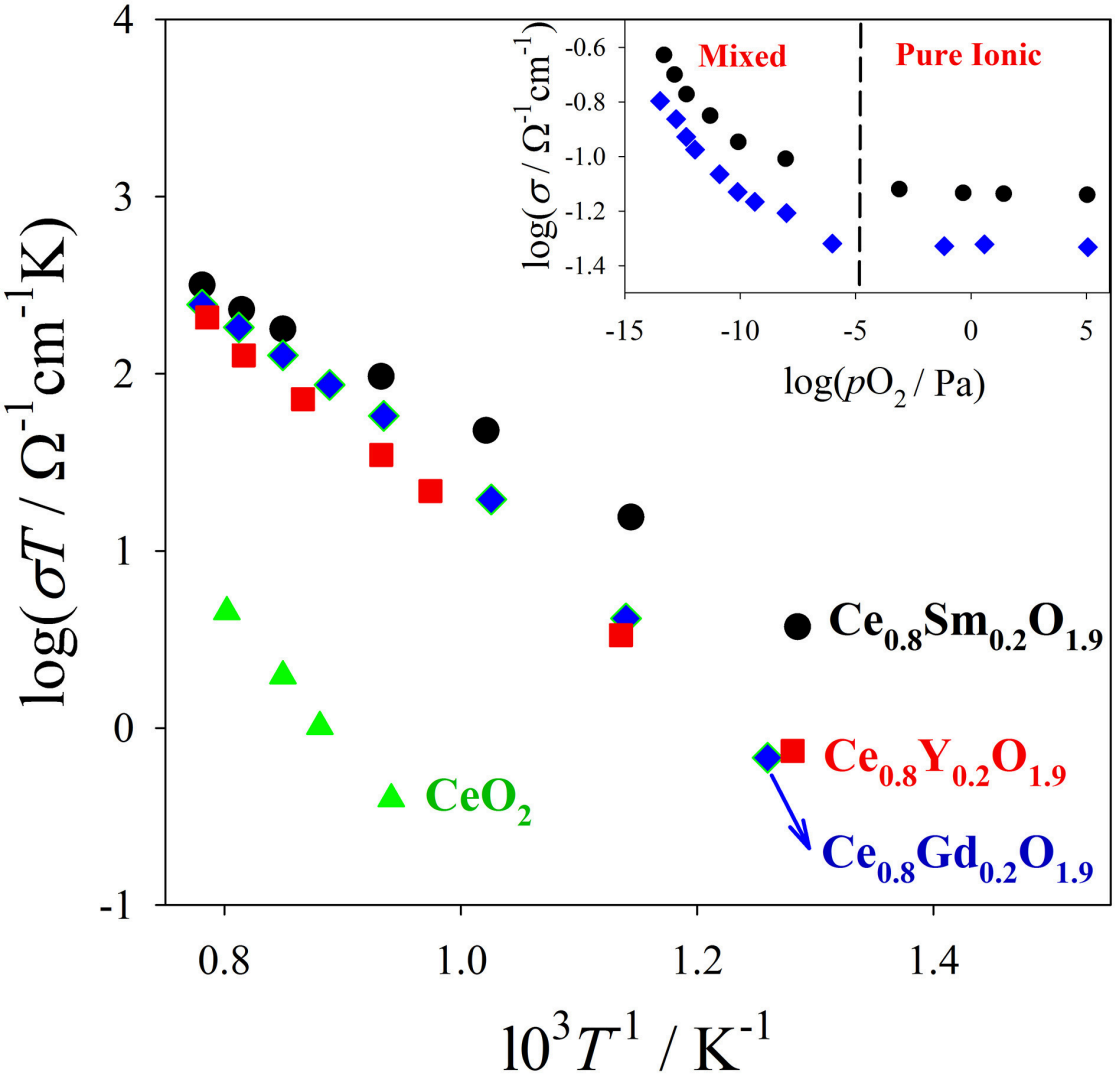
Bulk ionic conductivity data of different Ce_0.8_RE_0.2_O_1.9_ solid solutions measured in air. In the inset are shown conductivity data collected at 800°C on Ce_0.8_Sm_0.2_O_1.9_ (black circles) and Ce_0.8_Gd_0.2_O_1.9_ (blue diamonds) vs. *p*O_2_. In the low *p*O_2_ range, also electrons contribute to charge transport (ionic/electronic mixed regime). Data from Eguchi et al. (1992).

In Figure 2 conductivity data (from Eguchi et al., 1992) on (Y, Gd, Sm)-doped CeO_2_ are plotted against *T*. Activation energies for conduction can be calculated fitting the Arrhenius plots (logσ*T* vs *T*^−1^) of Figure 2. The plots of the insets of Figure 2 (logσ vs. *p*O_2_*)* assess the electronic and ionic contributions to conductivity: at high *p*O_2_, σ is constant and fully ionic; conversely σ increases lowering *p*O_2_ by reason of additional electronic contribution. For application as a solid electrolyte in SOFC/SOEC it is important to assess the zone of *x, T* and *p*O_2_ where the equilibrium with the atmosphere brings to the formation of additional V_O_s according to equilibrium (2) because electronic conduction provokes undesired shortcuts between the electrodes.

A huge number of experimental and review papers focus on the transport properties of RE-doped ceria solid solutions as a function of temperature, oxygen partial pressure, composition and microstructure. Limiting to reviews (Jacobson, 2010) and (Goodenough, 2003), discussed the suitability of Ce_1−x_RE_x_O_2−x/2_ materials for SOFC applications while Inaba and Tagawa (1996) and Kilner (2008) and, more recently, Koettgen et al. (2018) reviewed their electrical conductivity.

Although data from different groups are highly scattered, as pointed out by Koettgen et al. (2018), some general trends emerge. As suggested by Equations (6, 7), isothermal ionic conductivity increases with x. However, this applies to light doping only. σ_i_ decreases above a critical concentration *x*_c_ depending on RE nature and temperature, as observed for Gd (Faber et al., 1989; Zhang et al., 2002, 2004; Zha et al., 2003), Sm (Zhan et al., 2001; Jung et al., 2002; Zha et al., 2003), Y (Wang et al., 1981; Faber et al., 1989; Zhang et al., 2004; Sato et al., 2009) and other dopants like La, Nd, Yb (Faber et al., 1989; Dikmen et al., 1999). The critical concentrations *x*_c_ reported in the literature are scattered and usually occur in the range *x*_c_ = 0.06-0.2, depending on RE and temperature considered (Koettgen et al., 2018). For example, maxima for Gd were observed by different authors at *x*_c_~0.10 (Steele, 2000), 0.15 (Zha et al., 2003), and 0.20 (Zhang et al., 2002).

**Figure 3:**
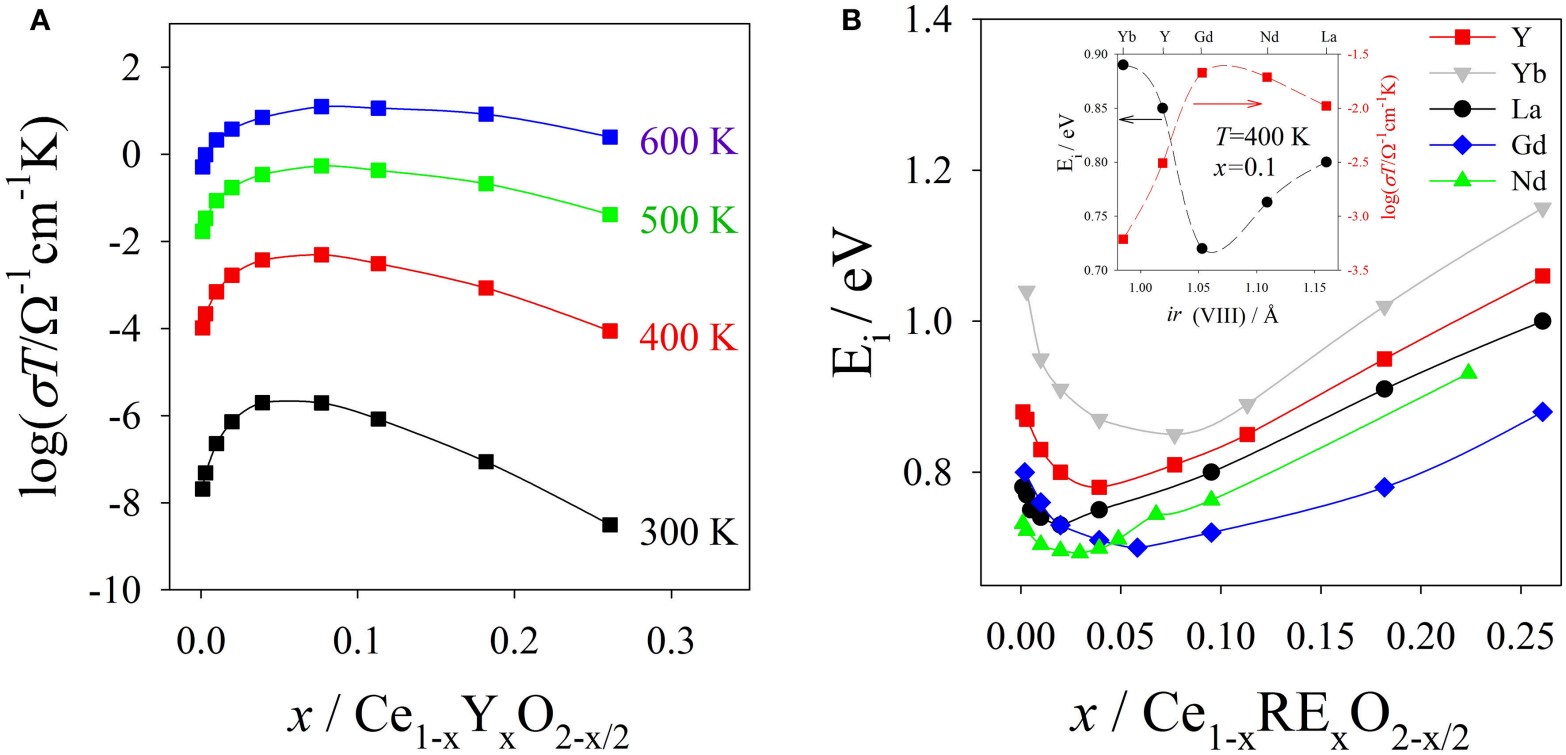
**(A)** Conductivity data of Ce_1−x_Y_x_O_2−x_ vs. composition *x* at different *T* values. **(B)** Activation energies E_i_ vs. *x* for Ce_1−x_RE_x_O_2−x_ solid solutions. In the inset are shown the E_i_ values in the (300 < T < 600 K range) together with σ_i_ values at 400 K and *x* ≈ 0.10 as a function of the ionic radii of the 8-fold coordinated RE^3+^ ions. Data from Faber et al. (1989).

As an example, σ_i_(*x*) vs. *T* registered for Ce_1−x_Y_x_O_2−x/2_ are reported in Figure 3A (data from Faber et al., 1989). Isothermal conductivity maxima appear around *x* ≈ 0.10, at which only 2.5% of the oxygen sites are vacant. The measured activation energies also depend on *x* and show minima around the same *x* values (Kilner, 2008). In Figure 3B the E_i_ values as a function of *x* are displayed (data from Faber et al., 1989).

E_i_ and σ_i_ also change by moving across the lanthanide series: the highest σ_i_ is observed for Gd and Sm (Eguchi et al., 1992; Chen et al., 1997; Omar et al., 2006; Zajac and Molenda, 2008), while σ_i_ reduces for lighter and heavier RE elements. The inset of Figure 3B shows E_i_ and σ_i_ at *T* = 400 K and x ≈ 0.10 as a function of the RE ionic radius (data from Faber et al., 1989).

To rationalize the bell curves of Ei and σ_i_ vs. the ionic radii, several authors evidenced the role of ionic radii (*ir*) mismatch between Ce^+4^ and RE^+3^. It should be noted that, for plotting purposes, in Figure 3B we used the ionic radii of the 8-fold-coordinated Re ions (Shannon, 1976). However, the correct coordination to calculate the “mismatch” is matter of debate and it will be discussed in section Lattice Parameters.

Following the “mismatch” idea, too small and too big RE^3+^ cations cause large structural distortions depressing conductivity. Codoping with a suitable combination of small and big cations has been proposed to minimize elastic strain and improve conductivity (Yoshida et al., 2001; Wang et al., 2004; Omar et al., 2006, 2007; Kilner, 2008; Zajac and Molenda, 2008). For example, it has been observed that Sm and Nd-co-doped ceria is more conducting than single Gd-doped ceria at 550°C (Omar et al., 2008); on the other hand, Ce_0.90_Lu_x_Nd_y_O_1.95_ is less conductive than Ce_0.90_RE_0.10_O_1.95_ with RE = Y, Sm, Gd (Omar et al., 2006).

Conductivity measurements display complex behaviors moving along the *x* and *r*_i_ coordinates. Actually, ionic conductivity is a macroscopic quantity that sums up (and average) a plethora of elementary migration processes each one involving a jump of one oxygen ion from a lattice site to an empty vacant site around. Thus, the local structure rather than the average one does influence these processes. As pointed out by several authors, part of the V_O_s could be excluded from the diffusion process (at least within a certain temperature range) because of RE-V_O_s defect clustering. V_O_s involved in defect clusters should be thermodynamically more stable than the remaining ones thus hindering or reducing the jump frequency toward them. On the other hand, the activation energy for diffusion could depend on the local structure around the jumping coordinate and also include a term for the interaction of the vacancy with other point defects (Kilner, 2008). Koettgen and coworkers identify two phenomena: “trapping” and blocking. In the former the RE distribution affects the initial and the final oxygen sites energies differently; migration energy is therefore different for forward and backward jumps. In the latter, forward and backward energies are equal but dopants affect the transition state energy (Koettgen et al., 2018).

To apply these ideas to real ceria-based phases it is fundamental to map the RE and V_O_s distribution as a function of RE nature, concentration and temperature. This implies knowledge of the structure at different length scales using different experimental techniques spanning from diffraction to spectroscopy, microscopy and magnetic resonance. This is the core of the present review and some findings are presented in the next section. Then, coupling theoretical calculations to experimental findings should allow estimating also the energies at work on defect clusters formation and on oxygen migration paths (see section Atomistic Modeling Methods of Doped Ceria for some details).

## Experimental structural probes

This section aims at gathering the main experimental findings on structure and defects in doped ceria. First we consider undoped ceria, with a focus on the structure evolution induced by oxygen non stoichiometry and consequent defect structures. Then we review the effect of doping in CeO_2_-RE_2_O_3_ systems, highlighting stability ranges and nature of the different phases involved. We will introduce local scale investigations with different probes and the postulation of defect models, moving toward longer scale defects and discussing how crystallite size and synthesis route affect structure and defects. Eventually the discussion will move toward fuel cells' operating conditions.

### Undoped ceria

Cerium oxide exhibits fluorite structure: cubic, space group *Fm*-3*m*, with Ce and O in special positions, namely Ce in 4a (0, 0, 0) and O in 8c (¼, ¼, ¼). Ceria is used as reference standard material by NIST. Fluorite is stable over a wide range of *T* and oxygen non stoichiometry δ. No phase transformation was found down to 2 K by neutron powder diffraction (Coduri et al., 2012a), while high temperature promotes the splitting of O into two different sites consistent with a disordering along <111> direction (Yashima et al., 2006).

When CeO_2_ is reduced to CeO_~1.7−1.8_, a disordered, non-stoichiometric, fluorite-related phase (α) forms (Bevan and Kordis, 1964; Trovarelli, 2002). By further increasing δ, a number of fluorite related superstructures were observed, lowering the cell symmetry owing to vacancy orderings along the fluorite <111> direction, consistently with high temperature studies (Yashima et al., 2006). A comprehensive description of the superstructures in CeO_2−δ_, down to CeO_1.66_, is given by a single crystal neutron diffraction study (Kuemmerle and Heger, 1999).

Though still fluorite, the atomic scale structure can be different. An example for CeO_2_ is given in Mamontov and Egami (2000). Using Pair Distribution Function (PDF), they probed Frenkel-type defects in octahedral sites. These defects, which can be removed by high-temperature treatment, are related to the oxygen storage capacity of ceria. Hence, the structure is fluorite, but the application is driven by defects that alter, locally, the fluorite structure.

More in general, fluorites look simple, but they are not. Fluorites accommodate high concentration of lattice defects, especially in terms of V_O_s (Kim, 1989; Malavasi et al., 2010). Their wide temperature and stoichiometric stability can hinder the real local atomic arrangement, which in complex materials is often the one at the basis of the application (Egami and Billinge, 2003) This makes fluorite more interesting, but more challenging, to control and investigate.

### Doped system: long range structure

#### Phases and solid solutions

As described above, doped ceria can serve as electrolyte thanks to the ability of fluorite to host high concentration of mobile oxygen vacancies. Then, it is useful to quantify the maximum amount of dopant (*x*_max_) that enters fluorite without changing its structure.

**Figure 4:**
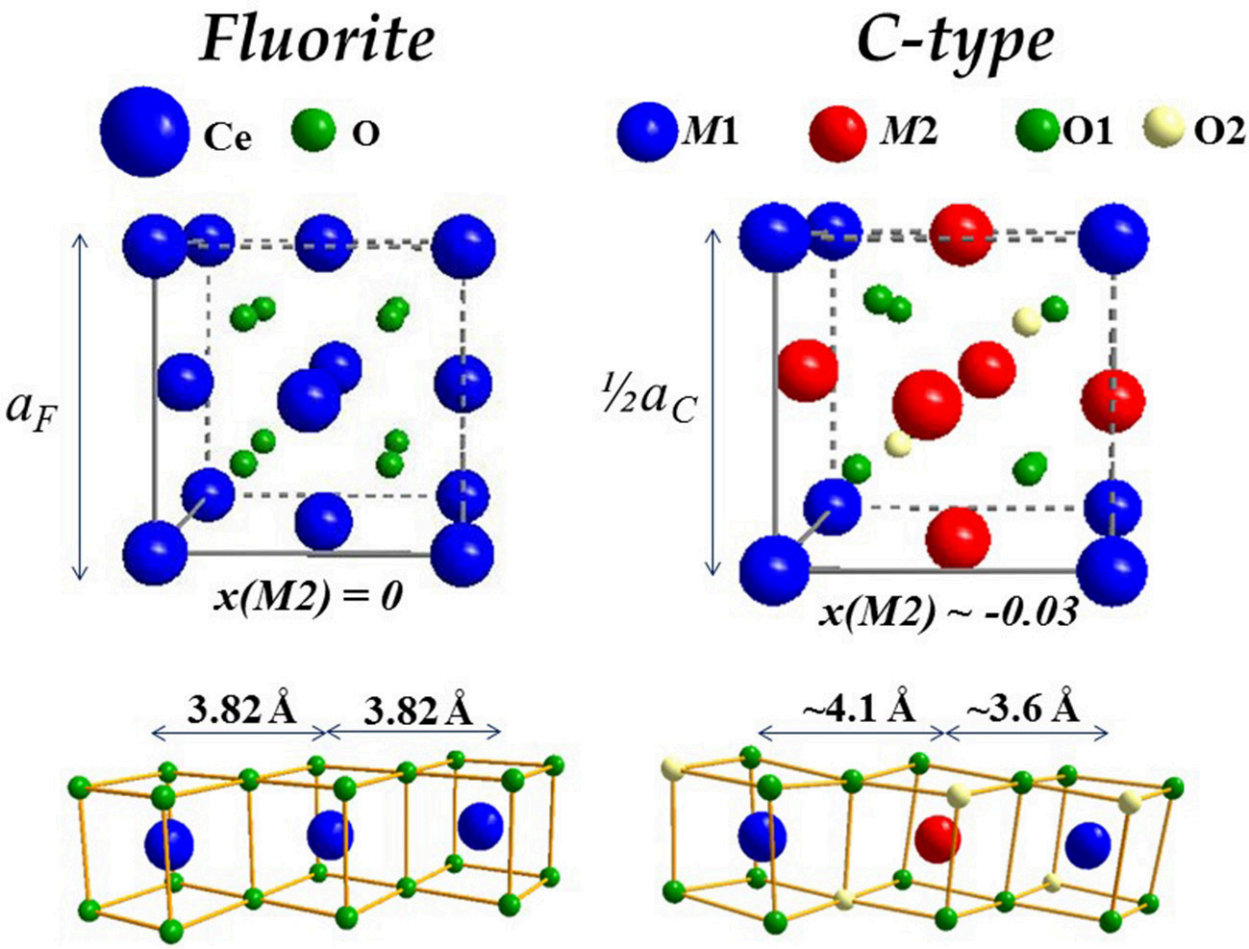
Sketch of fluorite **(Left)** and C-type **(Right)** structures, the latter displayed as octant of full cell to facilitate comparisons. The corresponding M-M connectivity is displayed below.

Before discussing *x*_max_ values, it is useful to introduce the dopant oxide phases. Cubic C-type, monoclinic B and hexagonal A are the three forms of sesquioxides (RE_2_O_3_) observed at ambient conditions. In particular, Nd and La form A-type, all other dopants lead to C-type, even though B-type was observed at RT after high temperature annealing for Eu (Ainscough et al., 1975), Gd (Grover and Tyagi, 2004), and Sm (Mandal et al., 2006; Artini et al., 2015; Coduri et al., 2018) and even in Y_2_O_3_ nanoparticles (Guo et al., 2006). More details about sesquioxides' structures are reported elsewhere (Adachi and Imanaka, 1998). Among sesquioxides, C-type (s.g. *Ia*-3) is the one closest to fluorite, the former being a structural distortion of the latter. In F and C-type the cell origin is positioned on *m*-3*m* (Ce site) and -3 (empty site) sites, respectively, a rigid shift of atomic positions is necessary to overlap the two structures. Atomic positions in the

two phases are summarized in Table 1 and sketched in Figure 4, where the full fluorite unit cell (*a*) is compared to one octant of the C-type phase (*b*). As a consequence of V_O_s ordering, when moving from fluorite to C-type the lattice parameter doubles, one cation coordinate (*x* of the M2 site) and four atomic coordinates of the O site move out of the special position. Whereas cations in fluorite lie at the center of an ideally perfect cube formed by 8 coordinated oxygen ions, in C-type they are 6-fold coordinated and the cation on the 24*d* site moves away from the center because of electrostatic repulsion with V_O_s. This affects significantly the distribution of interatomic distances, especially for M-M pairs (see Figure 4, bottom), which split into two well separated sets of distances in C-type compared to a single M-M distance in fluorite. Moving from F to C-type produces also the disordering of M-O pairs.

**Table 1:** Crystallographic relationships between fluorite and C-type phases of Gd-doped ceria compounds.

	**Fluorite**	**Fluorite (C-type setting)**	**C-type**
M1	4*a* (0, 0, 0)	8*b* (¼, ¼, ¼)	8*b* (¼, ¼, ¼)
O1	8*c* (¼, ¼, ¼)	48*e* (3/8, 1/8, 3/8)	48*e* (*x, y, z*)
M2	=M1	24*d* (0, 0, ¼)	24*d* (*x*, 0, ¼)
O2	=O1	16*c* (3/8, 3/8, 3/8)*	16*c* (*x, x, x*)*

*Empty site in Gd_2_O_3_, site occupation proportional to Ce amount.

**Figure 5:**
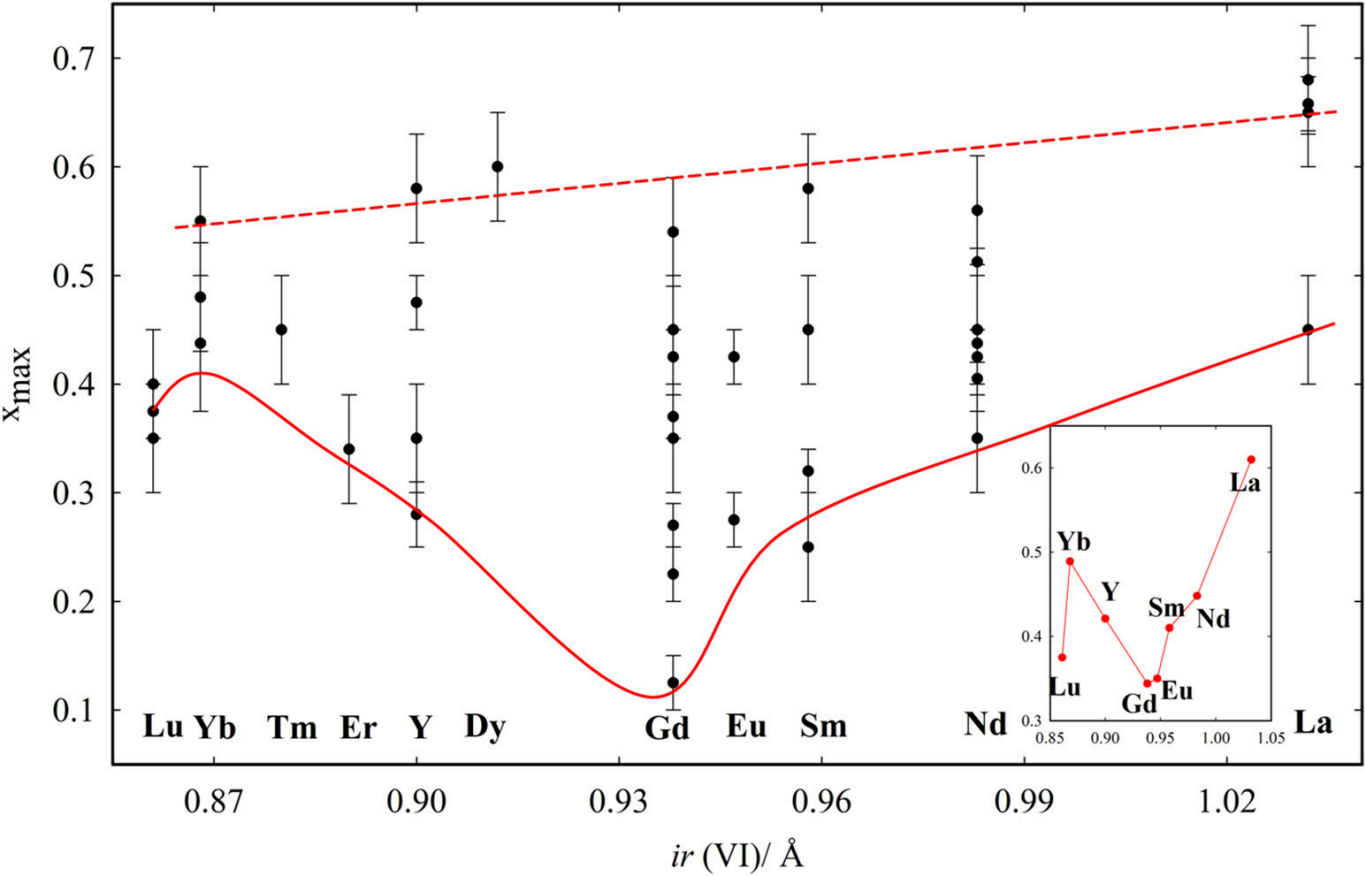
*x*_max_ values reported in the tabulated data from the literature. Red lines are guides to the eye, related to the largest and smallest *x*_max_ observed for each composition. The inset reports the average *x*_max_ values obtained for each dopant with at least two entries.

The *x*_max_ values from literature are listed in Table 2, with references, and plotted in Figure 5 to have an overview. We define *x*_max_ as the intermediate value between the last fully fluorite and the first non-fully fluorite dopant concentration *x*, the esd being half of step between the two compositions. Surprisingly, *x*_max_ changes significantly with the study, especially for intermediate size dopants. For Gd^+3^, *x*_max_ ranges from 0.125 (Nakagawa et al., 2001) to 0.54 (Bevan and Summerville, 1979). The latter (dashed red line in Figure 5) reports the highest *x*_max_ values for each dopant. It was proposed (Grover and Tyagi, 2004) that such high *x*_max_ values are a consequence of quenching from 1600°C and therefore might not be representative of the material at ambient. Also, above 1500°C mixed oxides can undergo a reversible transformation from solid solution to separated oxides. See Wallenberg et al. (1989) and references therein for details.

**Table 2:** Ionic radius for different coordinations, x max with corresponding reference, and average of the *x*_max_ values for each dopant.

	**i.r.(VI)**	**i.r.(VII)**	**i.r.(VIII)**	**Xmax**	**References**	**Average xmax**
Lu	0.861	–	0.977	0.35(5)	Grover et al., 2008	0.375(25)
				0.375(25)	Malecka et al., 2008
				0.40(5)	Artini et al., 2016
Yb	0.868	0.925	0.985	0.48(5)	Bevan and Summerville, 1979	0.489(57)
				0.55(5)	Mandal et al., 2007
				0.438(63)	Coduri, 2013
Tm	0.99	-	0.994	0.45(5)	Mandal et al., 2007	–
Er	0.89	0.945	1.004	0.34(5)	Horlait et al., 2011	–
Y	0.90	0.96	1.019	0.58(5)	Bevan and Summerville, 1979	0.421(130)
				0.475(25)	Chavan et al., 2004
				0.28(3)	Coduri, 2013
				0.35(5)	Satake et al., 2010
Dy	0.912	0.97	1.027	0.60(5)	Bevan and Summerville, 1979	–
Gd	0.938	1.00	1.053	0.54(5)	Bevan and Summerville, 1979	0.344(133)
				0.125(25)	Nakagawa et al., 2001
				0.27(2)	Scavini et al., 2015
				0.225(25)	Kossoy et al., 2013
				0.425(25)	Banerji et al., 2009
				0.35(5)	Artini et al., 2012
				0.37(2)	Chen and Navrotsky, 2006
				0.45(5)	Grover and Tyagi, 2004
Eu	0.947	1.01	1.066	0.275(25)	Shuk et al., 2000	0.35(11)
				0.425(25)	Mandal et al., 2006
Sm	0.958	1.02	1.079	0.58(5)	Bevan and Summerville, 1979	0.41(13)
				0.45(5)	Mandal et al., 2006
				0.45(5)	Nitani et al., 2004
				0.32(2)	Coduri et al., 2018
				0.25(5)	Artini et al., 2015
Nd	0.983	1.09	1.109	0.56(5)	Bevan and Summerville, 1979	0.448(60)
				0.513(13)	Chavan et al., 2005
				0.425(25)	Ikuma et al., 2005
				0.35(5)	Hagiwara et al., 2009
				0.438(63)	Coduri, 2013
				0.45(5)	Zhang et al., 2016
				0.405(15)	Horlait et al., 2011
				0.45(5)	Nitani et al., 2004
La	1.032	1.10	1.16	0.68(5)	Bevan and Summerville, 1979	0.61(11)
				0.658(25)	Andrievskaya et al., 2011
				0.45(5)	Bellière et al., 2006
				0.65(5)	Wilkes et al., 2003

*x_max_ is defined as the intermediate point between the last fluorite and first non-fluorite composition observed. The esd in bracket is half of the composition step between two consecutive points*.

The similarity between the F and C phases can be at the origin of the wide dispersion of *x*_max_. The structure is routinely defined trough XRD. Diffraction patterns of C-type differ from fluorite by the appearance of additional superstructure peaks, hard to resolve from background if data are noisy, especially when the C-type distortion is small (*x*(M2) close to 0) and superstructure peaks low in intensity. Moreover, approaching the F to C-type transition, superstructure peaks are broader (Coduri et al., 2013a), thus leading to overestimation of *x*_max_. This problem becomes even more important in nanoparticles for the further peaks broadening. In these cases, a C-type cell can be misinterpreted as a pseudo-fluorite cell, with lattice parameter *a*_*f*_ = *a*_*c*_/2, where *a*_*c*_ is the C-type lattice parameter.

Different *x*_max_ values might arise also from the usage of different notations for dopant concentration, which sometimes is defined as RE_2_O_3_ molar content (see Andrievskaya et al., 2011) rather than REO_1.5_. We recommend to define explicitly the relative cation stoichiometry, using formulas such as Ce_1−x_RE_x_O_2−y_.

The effect of the dopant on *x*_max_ was firstly investigated by Chavan and Tyagi (2005), who compared different dopants with *x* = 0.50. They noticed that La and Nd maintain fluorite structure, while the ionic size contraction of the dopant leads to the formation first of C-type phase, then to biphasic systems. Similar results were observed in Coduri (2013) and Horlait et al. (2011). The dopant dimension affects the minimum values of *x*_max_. The solid red line in Figure 5 is a guide to the eye indicating the minimum *x*_max_ values. The average *x*_max_ values are reported in the inset. *x*_max_ varies smoothly with the dopant size, reaching a minimum for Gd. Lu is out of the trend, even though three independent reports gave similar results. This was explained in Artini et al. (2016) by considering that the Lu^+3^ ions are so small that they can actually fit the size of the host Ce^+4^ retaining their full coordination. As a consequence, Lu^+3^ behaves as larger lanthanoids and the V_O_ is closer to Ce^+4^ rather than Lu^+3^. It is suggestedt that *x*_max_ is ruled not only by the size mismatch between Ce^+4^ and the dopant, but also by compressibility, i.e., the ability of the dopant to accommodate to a pressure change (induced by size mismatch) by expanding or contracting the unit cell. Compressibility scales monotonically with the ionic radius, thus explaining the largest *x*_max_ for La^+3^ among lanthanides. A size mismatch larger than 10% was proposed to be a condition to inhibit the formation of homogeneous C-type phase (Artini et al., 2017).

Eventually, one can expect a large *x*_max_ to come from a low stability of the corresponding C-type phase. Nd and La, which are often observed to have *x*_max_ ≥ 0.5, tend to form A-type phase, rather than C-type, which is less akin to fluorite. The transformation from C-type to other sesquioxides was proposed (Horlait et al., 2011) to occur when the average ionic size exceeds a threshold value, which can be reached only for the biggest cations.

#### Lattice parameters

**Figure 6:**
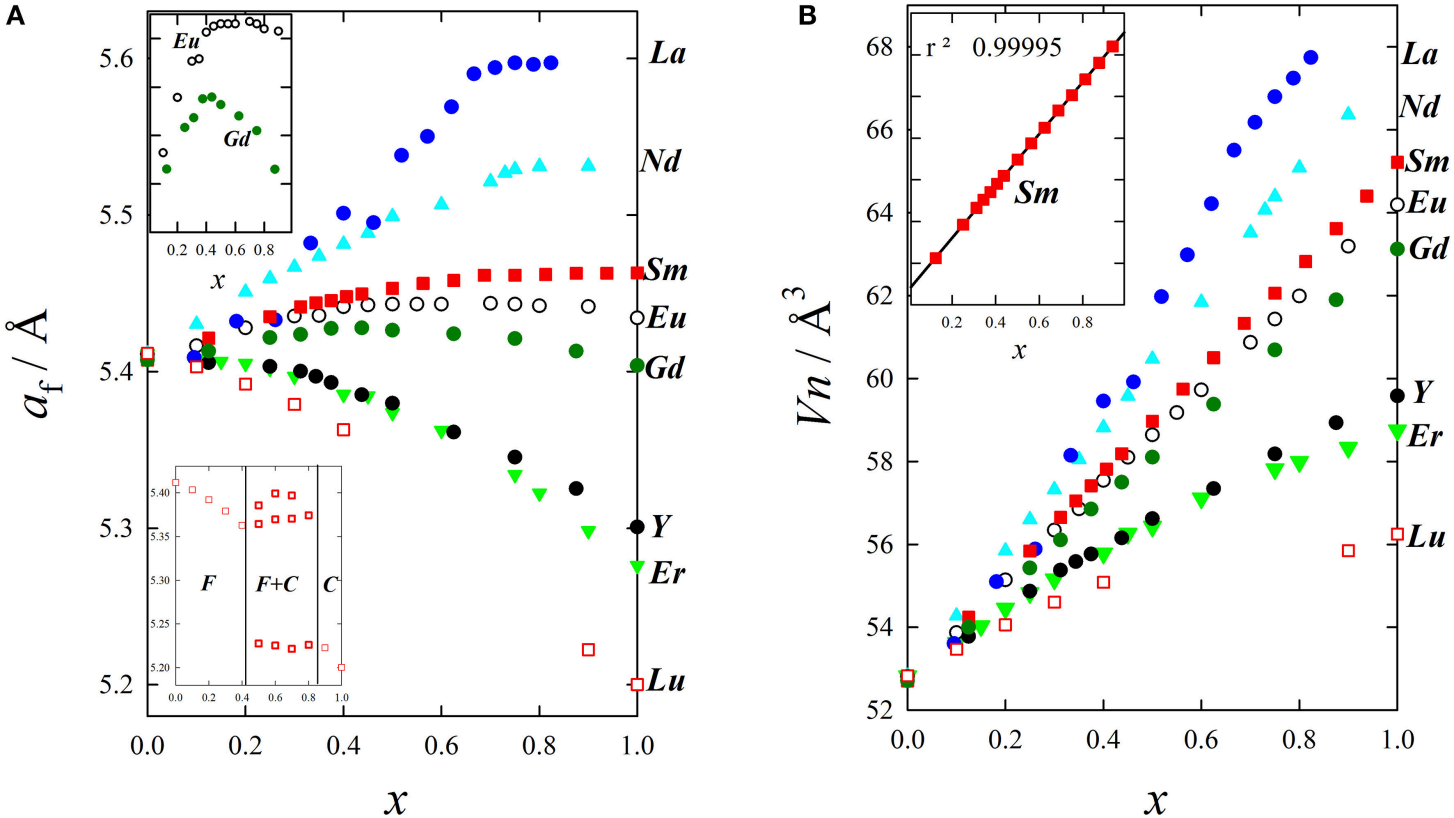
Evolution upon doping of **(A)** lattice parameter and **(B)** mean atomic volume for La (Andrievskaya et al., 2011), Nd (Horlait et al., 2011), Sm (Coduri et al., 2018), Eu (Mandal et al., 2006), Gd (Scavini et al., 2015), Y (Coduri et al., 2013a), Er (Horlait et al., 2011), and Lu (Artini et al., 2016).

Figure 6A shows the evolution of *a*_*f*_ for wide compositional ranges with different dopants. It is clear that doping produces expansion or contraction depending on the size of the dopant ion, and that Vegard's law is not followed. Excluding Tb and Pr, characterized by a mixed valence state (Nitani et al., 2004; Martinez-Arias et al., 2005; Coduri et al., 2014) and therefore to be dealt with separately, expansion is observed from La to Gd, while Y and other trivalent lanthanoids induce contraction. Considering the ionic radii reported by Shannon, 1976, (see Table 2) the volume change upon doping fluorite is consistent with the insertion of a 7-fold coordinated trivalent dopant. As an example, ionic radius (*i.r*.) for Y^+3^(VII) is 0.965 Å, slightly smaller than Ce^+4^(VIII) (0.97 Å); therefore Y-doping for Ce induces a feeble lattice contraction. Y^+3^(VIII), being 1.02 Å in size, would instead expand the cell. As not all RE have a known *i.r*. for coordination (VII), *x*-axis in Figure 5 reports coordination VI.

The size effect of different dopants is generally evaluated exploiting the lanthanide contraction. Whether the chosen coordination number (CN) is 6 (Artini et al., 2017), 7 (Coduri et al., 2012b, 2014; Shirbhate et al., 2016), or 8 (Eguchi et al., 1992; Balazs and Glass, 1995; Yoshida et al., 2001; Yashima and Takizawa, 2010), lattice contraction is experienced when moving from La to Lu.

Yet, the absolute value of the size mismatch strictly depends on the chosen coordination for the dopant. For example, Ce^+4^(VIII) has ionic radius 0.97 Å, which nearly corresponds to Lu^+3^(VIII) (0.977Å), Dy^+3^(VII) (0.97 Å) or Sm^+3^(VI) (0.96 Å). As different dopants with the same concentration can have different CN, the actual *i.r*. of the dopant can change non-monotonically within the lanthanide series. This should be taken into account when making comparison based on *i.r*.

If the amount of dopant rather than its nature is varied, deviations from Vegard's law are evident. Their origin is often source of debate. Horlait et al. fitted the compositional dependence of lattice parameters using a quadratic function. Parameters are tabulated in Horlait et al. (2011). Deviations from the Vegard's law were correlated to the presence of vacancies and consequent variation in CN (Nakamura, 2010). In Giannici et al. (2014) and Artini et al. (2016) the volume change upon doping was considered as the balance between the contraction arising from the formation of oxygen vacancies, size mismatch and local scale interactions.

A possible explanation for this behavior is that the unit cell's total number of atoms varies upon doping because of the formation of V_O_s. If the cell volume is normalized against the total number of atom for each composition, given the atomic mean volume *Vn* (Zen's law, Zen, 1956), linear trends are obtained within the range corresponding to the solid solution (Artini et al., 2016), as shown in Figure 6B.

Because the application as ionic conductor limits the interest in doped ceria to fluorite phases, in which all ions lie in special positions, there are no structural degrees of freedom other than lattice volume and Atomic Displacement Parameters (ADPs). ADPs in fluorite increase significantly with doping (Scavini et al., 2012; Coduri et al., 2013a, 2018) and scale with the size mismatch with the dopant (Yashima and Takizawa, 2010; Coduri et al., 2012b). As already observed in Argyriou (1994) and Scavini et al. (2010), the thermal evolution of ADPs, compared to a reference standard, is an effective tool to probe disorder. Disorder was evidenced, but other approaches are required to map defect-induced structural changes at the local scale.

### Local scale

One of the first local scale investigations came with a pioneering neutron powder diffraction study (Anderson and Cox, 1983), where different dopants-vacancy clusters were tested to model diffuse scattering. Though limited by instrumental setup, they demonstrated the existence of local ordering breaking the symmetry of fluorite, proposing the formation of clusters of dopant and V_O_s. Yet the structural information was rather qualitative. In the last decades a number of local structure investigations were carried out. This section will provide a survey, technique by technique, for unveiling the defect structure in doped ceria.

#### X-ray absorption spectroscopy

**Figure 7:**
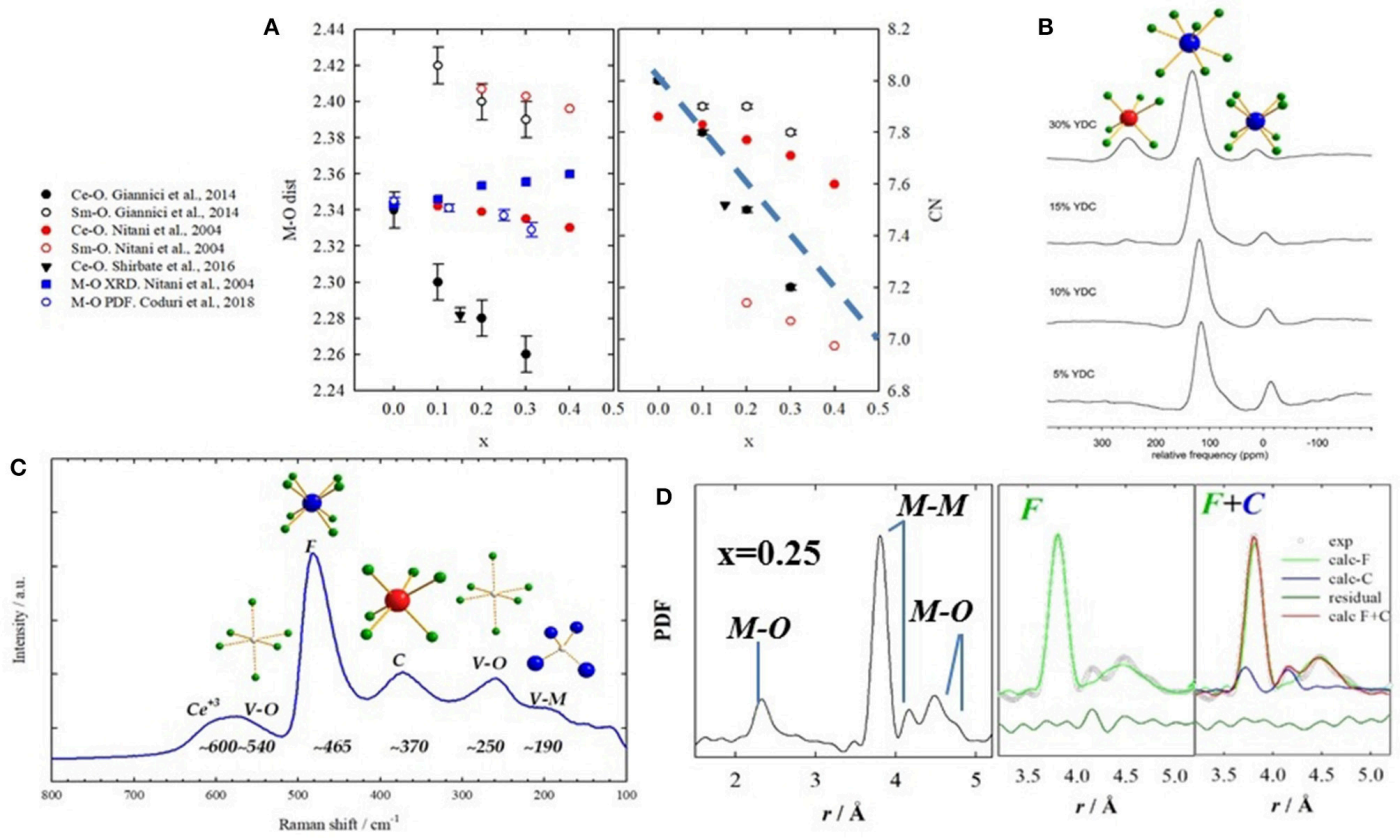
**(A)** Interatomic M-O distances (left) and coordination numbers (right) obtained by EXAFS on Sm-doped ceria from different works, listed on the left side. Full symbols stand for M = Ce, empty symbols for M = Sm. Black circles refer to data from Giannici et al. (2014); red circles from Nitani et al. (2004); black triangle from Shirbhate et al. (2016). Interatomic distances are also compared with XRD and PDF, in full and empty blue symbols, respectively. **(B)** NMR spectra of Y-doped compounds showing different coordinations to O. Labels stand for the 6-, 7-, and 8-fold coordinations. Reprinted with permissions from Kim and Stebbins (2007). © 2012 American Chemical Society. **(C)** raman spectrum for x(Gd) = 0.375 with labels representing the coordinations involved. Data from Coduri et al. (2017). **(D)** Experimental PDF for x(Sm) = 0.25 with corresponding atom pairs (left). Fit of the same curve using single fluorite (green) and mix of fluorite and C-type (blue) on the right hand side. Data from Coduri et al. (2018).

The advent of new generation synchrotrons allowed EXAFS studies to be performed routinely providing a direct approach to inquire local scale in doped ceria compounds. Hormes et al. (2000) investigated different dopants, confirming that Ce and RE maintain their +4 and +3 state, respectively. Exceptions with mixed +3/+4 valence state are Pr (Hormes et al., 2000; Nitani et al., 2004) and Tb (Martinez-Arias et al., 2005). Ohashi et al. (2005) used EXAFS to probe the local scale of Gd-doped ceria up to *x* = 0.30. They observed that Gd-O distances are larger than Ce-O, and that they shrink with doping even though the unit cell expands. They proposed the formation of clusters made of a V_O_ and two dopant ions as well as the relaxation of oxygen ions toward the induced V_O_ to explain the M-O contraction. A number of similar investigations followed (Yamazaki et al., 2000, 2002; Nitani et al., 2004; Deguchi et al., 2005; Wang et al., 2006; Kossoy et al., 2013; Giannici et al., 2014). A common outcome is that Ce-O and RE-O distances are different, the latter scaling with the size of the dopant. In general, the coordination number (CN) of Ce is higher than the dopant's. An exception was reported by Giannici et al. (2014), who observed fully 8-coordinated Sm with V_O_s as nearest neighbor (NN) of Ce. A lower CN(Ce) was noticed also in Shirbhate et al. (2016), but they only investigated the Ce-edge. These results conflict with EXAFS results in Nitani et al. (2004), which found a larger CN for Ce^+4^ than Sm^+3^ (see Figure 7A. Interestingly, atomistic calculations show that Sm^+3^ is nearly as stable as NN or NNN of the V_O_ (Nakayama and Martin, 2009; Hooper et al., 2010).

All investigations agree that CN decreases with doping together with the oxygen concentration. As the ionic size mismatch between host Ce^+4^ and dopant has been retained to affect transport properties (Eguchi et al., 1992; Balazs and Glass, 1995), codoping using two different RE ions with weighted ionic size similar to Ce^+4^ has been proposed (Mori et al., 2002). Yoshida et al. (2001) investigated double doping with La and Y but found no synergic effect. Local distortions happen to be more important than the global lattice strain. A correlation between local structure and transport properties was observed: the more the V_O_s are distributed randomly, the higher the ionic conductivity.

Yamazaki et al. (2000) sought for more complex defect clusters. Comparing different dopants, they found clusters composed of 2–4 dopant ions, depending on the nature of the dopant and its concentration. A similar approach was followed in Giannici et al. (2014). Deguchi et al. (2005) tried to extract EXAFS signal up to M-M distances, but esd on the coordination number happened to be as large the CN itself. This poses a limit on the structure information extracted from absorption spectroscopy: are defect clusters composed of a few units of dopants and vacancies, or is the signal intrinsically related to the first neighbors?

#### NMR

Solid state NMR can resolve different coordination environments from different chemical shifts. All doped ceria compounds can be investigated through ^17^O resonance to inquire the O local environment, exploiting ^17^O in natural O. Except for diluted systems, in La-doped ceria NN La^+3^ are always bonded though a V_O_ (Heinmaa et al., 2010). Still, the study was limited up to *x* = 0.116, and no evidence of bigger clusters was found. Kim and Stebbins (2007) considered higher loadings using Y as a dopant. Doping induces different local environments not consistent with a random distribution of dopant ions and V_O_s. Interestingly, for *x* = 0.15, a small fraction of O coordinated to 3 Y and 1 Ce and 4 Y ions is observed. ^89^Y evidenced different CNs for Y. Most of Y ions are 7-fold coordinated. From x~0.25 a significant portion of Y(VI) was observed (see Figure 7B). This again suggests that V_O_s are not randomly distributed and that the dopant might keep the local environment as in the pure oxide. Anyhow, the structural information extracted out of NMR is necessarily limited to the NN shell. Information about longer range structure could be only speculated on the basis of the observed CNs.

#### Raman spectroscopy

Indications on the charge, size, and spatial correlation of defects may be offered by Raman spectroscopy. The Raman spectra of doped CeO_2_ have key signals sensitive to the distortions in coordination cages arising from formation of both intrinsic or extrinsic V_O_s. In addition, there are clearly distinct signals for 6-fold and 8-fold coordination environments (Nakajima et al., 1994). A sketch of a typical Raman spectrum for heavily doped ceria is given in Figure 7C.

The onset of the Gd-O vibration in 6-fold coordination was shown to match well the phase boundary between long-range F and C-type phases (Coduri et al., 2017). The appearance of this signal is not only dependent on dopant concentration, but also on crystallite size, pointing to different defect association states at different sintering times and/or temperatures (Taniguchi et al., 2009; Coduri et al., 2017). In fact, the same signal was also attributed to local RE_2_O_3_ domains within a fluorite phase (Banerji et al., 2009; Artini et al., 2015). To distinguish between isolated RE defects, C-type nanodomains or a longer-range order one can explore the dependence of mode frequency on ionic size (Artini et al., 2017).

The distortion induced by dopants on the long-range scale was shown among others by McBride et al. (1994) who studied the changes to the main fluorite signal, the F_2g_ symmetric vibration mode of the Ce-O bond in 8-fold coordination (Nakajima et al., 1994). With all the dopants they tested (La, Nd, Eu, Gd, Tb, Pr) they attributed a red-shift of up to 5 cm^−1^ to the separate contributions of lattice expansion and V_O_s. They showed that after accounting for lattice expansion through the Grüneisen parameter the strictly trivalent dopants (La, Nd, Eu, Gd) actually produce a positive frequency shift related to their extrinsic V_O_s. This positive shift was then found in Sm-doped CeO_2_ (Artini et al., 2015) at the boundary between F and RE-rich phases. In nanopowders, size distribution and consequently inhomogeneous strain affects the asymmetric broadening of the F_2g_ signal, which can be described semiquantitatively using a phonon confinement model (Dohčević-Mitrovi et al., 2006).

Besides markers of 6-fold and 8-fold coordination environments, Raman spectra between 510 and 600 cm^−1^ yield information on the association of extrinsic *RE*_Ce_′ and $V_{O}^{••}$ defects and their relative amount with respect to intrinsic V_O_s and Ce^4+^ reduction. In the case of Gd it was noted that large crystallite size and a higher concentration of dopant favor extrinsic defects (Taniguchi et al., 2009; Coduri et al., 2017). In particular, it was proposed that as Gd concentration and sintering temperature are increased REO_8_ clusters initially scattered in the bulk diffuse and cluster and trap V_O_s. The high-frequency shift of the band around 540 cm^−1^ (Taniguchi et al., 2009; Coduri et al., 2017), moreover, suggests a further step from a 1:1 to a 2:1 ratio between *Gd*′_Ce_ and $V_{O}^{••}$.

#### HRTEM

**Figure 8:**
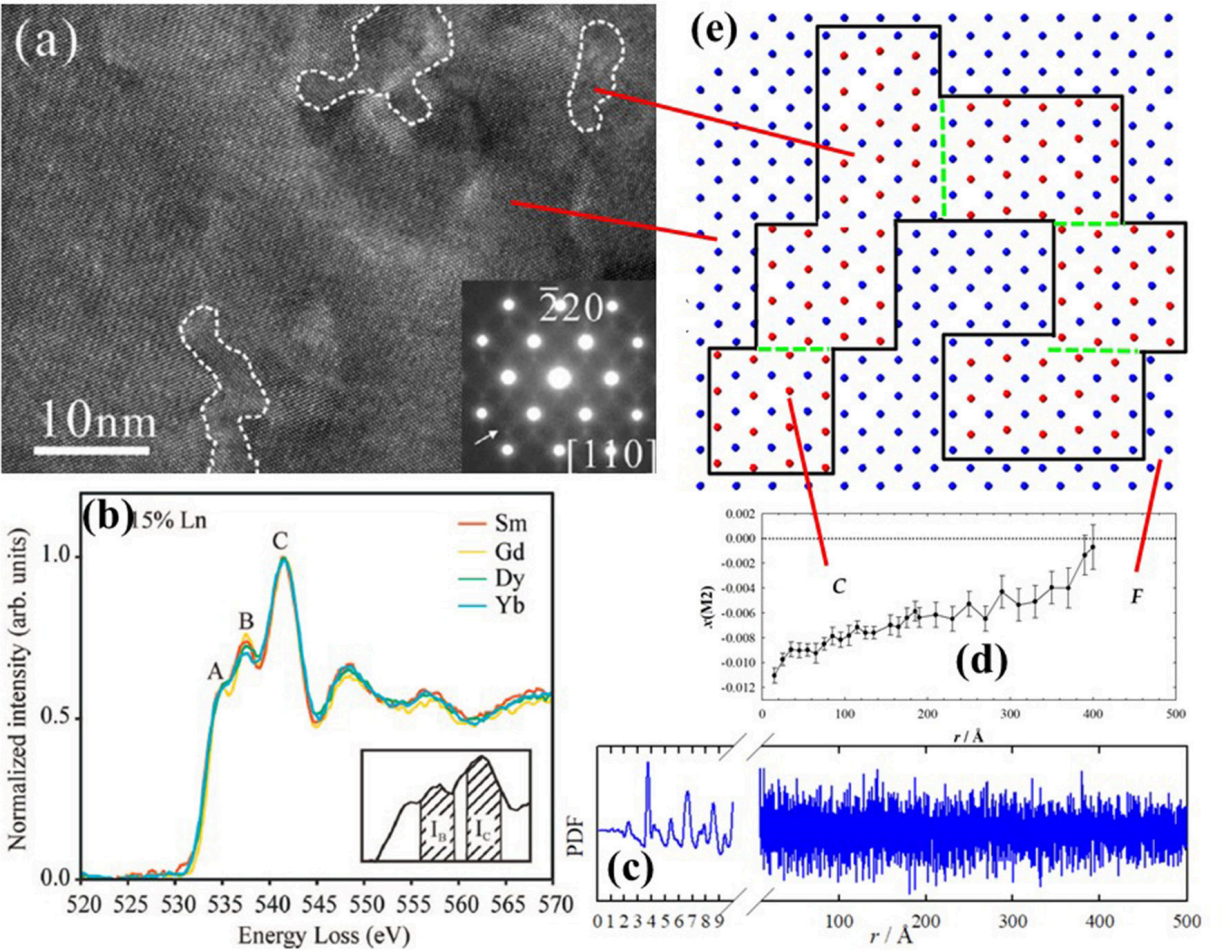
**(a)** Nanodomains within white dashed lines observed in x(Y) = 0.25 by HRTEM and corresponding SAED pattern. Reprinted with permissions from Li et al. (2012). © 2012 American Chemical Society. **(b)** Characteristic signals of EELS spectra taken at O K-edge for different dopants. Reprinted with permissions from Ou et al. (2008a). © 2008 American Physical Society. **(c)** Experimental PDF for x(Gd) = 0.344, and **(d)** corresponding evolution of x(M2) coordinate with interatomic distance *r*. Data from Coduri et al. (2017). **(e)** Sketch of cation arrangement as in Figure 4 within the basal plane (*z* = 0) representing C-type domains, enclosed by black solid line, embedded in a fluorite matrix. Green dashed lines stand for APBs. The concept is discussed in Coduri et al. (2013a).

In 2002 Mori et al. (2002) used HRTEM to show that the structure of Sm- and La-doped ceria is not homogeneous, but rather composed of nanometric domains with a different crystallographic phase (see Figure 8a). Further investigations by the same group confirmed the nanodomain structure for Dy (Mori et al., 2005), Gd (Mori and Drennan, 2006; Ye et al., 2008; Li et al., 2011), Y (Ou et al., 2006a; Li et al., 2012), Yb (Ou et al., 2006b) and Ho (Ou et al., 2008b). The domain size was proposed to be directly related to conductivity: larger domains trap more V_O_s thus affecting transport properties (Mori and Drennan, 2006). The structure of the domains was found to related be C-type (Ou et al., 2008a) using Selected Area Electron Diffraction (SAED) (see inset of Figure 8a). The space group *I*2_1_3 was then proposed (Ye et al., 2009). Li et al. (2011) revealed that dopant (and V_O_s) segregation occurs not only at grain interior, but also, to a lesser extent, at the grain boundaries.

Further information on defect structure can be gained through Electron Energy Loss Spectroscopy (EELS). Based on the work in Travlos et al. (2003), Ou et al. (2006a) showed that EELS collected at the O-K edge give a set of signals which can be correlated with the local scale structure. In particular, the characteristic peak at 543 eV reflects the V_O_s ordering (see Figure 8b). The ratio over the peak at 546 eV, whose intensity is not related to V_O_s, is a parameter to quantify the extent of C-type ordering. The method is now being used routinely (Chen et al., 2014; Lee et al., 2014). The limit of electron microscopy, as usual, is that the observed features may be non-representative of the full sample. This calls for other bulk methods to confirm the nanodomains structure and to quantify the size.

#### Total scattering

As discussed above, diffraction evidences the presence of disorder, but it is not able to describe it at the atomic scale. Disorder appears in diffraction data in the form of diffuse scattering which, coupled to the Bragg peaks in case of crystalline solids, gives the total scattering. A natural approach would be to study diffuse scattering in single-crystals, which unfortunately are not easily available in doped ceria. In powdered materials, diffuse scattering can be modeled directly in reciprocal space (Anderson and Cox, 1983). Debye Function Analysis (DFA) is another powerful tool to unveil the (disordered) structure of small nanoparticles. In particular, using DFA an atomistic model of the entire nanoparticle is fitted against the powder total scattering data (Cervellino et al., 2015; Bertolotti et al., 2016). To our knowledge, no papers exploiting DFA analysis of pure or doped ceria have been published up to now.

Total scattering data can be otherwise analyzed in real space after suitable corrections and Fast Fourier Transform through the atomic Pair Distribution Function (PDF) (Egami and Billinge, 2003). In 2012, the local atomic structure of ceria doped with different dopants (*x* = 0.25) was investigated using PDF (Coduri et al., 2012a,b). Neutron diffraction data were consistent with the simple relaxation of O ions toward the dopant-induced V_O_ (Coduri et al., 2012a), confirming the model proposed by experimental studies (Ohashi et al., 2005) and simulations (Inaba et al., 1999). The larger the size mismatch with Ce^+4^, the larger the structural distortion on the O sites.

Yet, X-ray PDFs is mostly sensitive to distances involving metals (M) ions rather than O and cannot be modeled by simple V_O_-dopant relaxations. In particular, in the F structure, only one M-M NN distance should appear, while in the C-type there are two M-M distances. Still, the second peak occurs even in F solid solutions evidencing C-type local ordering (see Figure 7D). Hence, the first coordination shells up to ~6 Å are consistent with a mixture of fluorite and C-type environments. Different dopants experience a similar local scale: Gd (Scavini et al., 2012), La, Nd, Yb (Coduri et al., 2012b), Y (Coduri et al., 2013b), Sm (Coduri et al., 2018), Zr (Gateshki et al., 2007), Bi (Sardar et al., 2010), Pr and Tb (Coduri et al., 2014). Scavini et al. (2012) probed a continuum of structure evolution throughout the full CeO_2_-Gd_2_O_3_ system, consistently with the monotonic increase of C-type at the local scale. Anomalous scattering confirmed that the dopant ion is involved in that PDF peak (Allieta et al., 2011).

Except for the clear observation of a longer M-M distance not compatible with a fluorite arrangement, the above results are consistent with other local scale studies. The NN M-O distance shrinks upon doping even though a global expansion is observed (Figure 7A), as already evinced by EXAFS (Yamazaki et al., 2000, 2002; Deguchi et al., 2005). The same applies to the main M-M distance (e.g., for Gd- Deguchi et al., 2005 and Sm-doping Giannici et al., 2014). Still, a gap exists between the local scale investigations and microscopy evidences of wider scale ordering.

When studying NN interactions, EXAFS can be preferred to PDF as the latter is not element sensitive and because termination ripples, implied by the finite energy of the incoming radiation, affect mostly the NN peak. On the other hand, PDF provides structural information on a wide length scale (Figure 8c), depending on the instrument resolution (Qiu et al., 2004). A PDF investigation of Y-doped ceria throughout the F to C-type transformation (Coduri et al., 2013a) demonstrated that disorder is not limited to the local scale, it rather evolves on a larger scale. Monitoring the evolution of *x*(M2) with the radial distance (Figure 8d and Table 1) allowed direct quantification of the C-type distortion. In this model, C-type domains are embedded in a fluorite matrix and their size increases with *x* until complete transformation to C-type. Details on the data analysis strategy were reported in Checchia et al. (2015). C-type domains as large as 12 nm were observed already for *x* = 0.25, when the structure is fluorite. The domain structure was confirmed by the observation of antiphase boundary (APB) in powder diffraction patterns of the first C-type samples formed upon doping, probed as systematic *hkl*-dependent broadening of superstructure reflections only. APBs occur when nucleation of low symmetry phase starts randomly on different lattice sites, with possible faults where domains meet. A similar picture applies to Gd- (Scavini et al., 2015; Coduri et al., 2017) and Sm-doping (Coduri et al., 2018). A mechanism involving the progressive orientation of small C-type droplets percolating into coherent nanodomains was proposed in Scavini et al. (2015). A sketch of percolated C-type domains in fluorite, with formation of APBs, is given in Figure 8E.

#### Final remarks

To conclude, the above techniques provide a set of complementary tools to gain a complete description of structure and defects. Diffraction tells whether the long range structure is fluorite or C-type, or both. If single phase, the presence (or not) of superstructure peaks defines a C-type (or fluorite) phase. Thus, XRD data have to be collected with proper counting statistics, paying attention to the region between the 2nd (200) and 3rd (220) diffraction peaks, where superstructure peaks might appear. Full scale patterns, often displayed in papers, are not useful to tell whether the structure is F or C. Powder diffraction is very powerful but it can be misleading. Noisy data would indicate that structure is fluorite rather C-type just because superstructure peaks might be hidden in the background. Finally, powder diffraction probes the long range structure but is nearly blind with respect to local orderings.

Raman spectroscopy is a powerful technique to probe local scale ordering as different bands can be assigned to different chemical coordinations. But the structural information provided by Raman is strictly local. The coexistence of fluorite-like (MO_8_) and C-type-like (MO_6_) signals is not a proof of full-scale segregation of the two phases. On the other hand, if only C-type signals are probed by Raman, the long range structure will be hardly fluorite.

As XRD and Raman are complementary structural probes widely available in research centers, it is recommendable to combine them as much as possible.

EXAFS is the technique to use to probe NN local interatomic distances for Ce and dopant. It has two main disadvantages. It requires synchrotron radiation, the access to which is generally awarded through a peer review process and not immediate. Expertise in EXAFS data analysis is necessary as large parameter correlations often make the significance of the results questionable. When studying NN distances EXAFS has still an edge on PDF, whose NN peak is not element resolved and often affected by termination ripples (Peterson et al., 2003).

EXAFS is also used to extract CNs. Proper CNs require spectra to be collected at both Ce and RE edges, and CNs to be constrained to the known stoichiometry in order to reduce correlations (Giannici et al., 2014). This could be the origin of the spread set of CN values in the literature. NMR is a very powerful tool to confirm CNs extracted from EXAFS, as it provides the relative abundance of the different coordinations. Unfortunately, to our knowledge, no combined NMR/EXAFS experimental report has ever been published.

HRTEM and PDF are the techniques providing information on larger length scales. The advantage of microscopy is that it can “see” domains, rather than extracting information out of a fit. Electron microscopes are also more accessible than synchrotron radiation. Yet, domains from HRTEM were investigated always by the same group. The observation of domains is not representative of the full material. Also, sample preparation is demanding, prevents the study of small particles and is commonly limited to ambient condition. On the contrary, being a bulk technique, PDF provides domain sizes representative of the full material. It is particularly suitable for doped ceria compounds as signal is proportional to the number of electrons *z*. Then, it is particularly sensitive to M-M pairs. Like EXAFS, PDF requires the use of synchrotron radiation, even though laboratory diffractometers for PDF are becoming increasingly common.

From the overview given in section Phases and Solid Solutions, it appears that some results are in contradiction. The compositional range for F and C-type as well as the existence of miscibility gap between the two phases change according to the study. CNs also are not fully reproducible. When moving from the routine characterization of a material to a more detailed study, it would be advisable to combine as many techniques as possible. That would be also helpful to comprehend the limitations of the techniques involved, as they would be applied exactly to same material.

### Defects arrangement from F to C-type

Nowadays different notations are used to describe the compositional region corresponding to the transition from fluorite to C-type. In Coduri et al. (2013a, 2018), Checchia et al. (2015), and Scavini et al. (2015) the authors defined as C^*^ the compositional region (x~0.3–0.5 depending on composition) characterized by the percolation of C-type domains. From a powder diffraction point of view, the C^*^ region is characterized by (i) non negligible concentration of APBs, i.e., anomalous broadening of superstructure peaks; (ii) a different linear dependence of atomic coordinate *x*(M2) with doping than the x(M2) trend in the “mature” C-type phase. This means that the structure is actually long range C-type, resulting from the percolation of C-type nanodomains in a fluorite matrix while large fluorite regions still exist.

Artini et al. (2015, 2016, 2017) described intermediate compositions for RE = Gd, Sm, Lu as a hybrid (H) phase between fluorite and C-type. The structure is generally C-type, but the coexistence of Raman modes typical of fluorite and C-type leads tothe picture of a hybrid structure, i.e., a sort biphasic system occurring at the local, or intermediate scale. This is similar to the C^*^ formalism, even though the H structure can be found in long range fluorite samples—the case of uncorrelated nanodomains in a long range fluorite structure does not apply to C^*^—and extend to a larger region in the C-type compositional range. The existence of H was found to depend on the size mismatch between dopant and host. If the size mismatch is too big, a single hybrid (C-type) phase is not stable, i.e., it cannot accommodate for both Ce and RE, and a long range phase separation into F and C-type is observed. Producing samples following the same protocol, Artini et al. (2016) observed long range F-C separation only for Tm, Yb, and Lu. Phase separations are observed for other dopants as well (Bevan and Kordis, 1964; Shuk et al., 2000). The reaction procedure does play a role in that, as either single fluorite or biphasic systems were obtained on the same material, respectively, after oxidizing and reducing annealings (Małecka et al., 2009).

### Size effect

Doped ceria can be successfully produced through a number of different synthetic routes. Most of early studies used solid state synthesis, involving reaction of CeO_2_ and RE_2_O_3_ mixed powders at high temperature with intermediate regrinding steps. Still, the observed local ordering with dopant oxide structure can either be an intrinsic characteristic or a consequence of an incomplete reaction.

The interest for surface effects called for the development of different synthetic routes to tune the morphology of the products. Manifold wet procedures were proposed (Van Herle et al., 1998; Reddy et al., 2009; Rezaei et al., 2009; Wang et al., 2010). These lead to nanoparticles that can be sintered afterwards to increase grain size and density. Moreover, wet methods are considered to lead to more homogeneous materials (Horlait et al., 2011).

The role of the synthesis is generally studied with respect to transport properties. Its relationship with defect structures or structure in general received less attention, with the exception of defects in nanometric samples, where surface effects become not negligible.

Tsunekawa et al. (2000) attributed the inverse relation of lattice expansion of CeO_2_ and crystal size to the stabilization of Ce^+3^ ions. In doped samples Ce^+3^ is often found to segregate at the surface of nanoparticles. Lee et al. (2014) observed that Y-doping for *x* > 0.09 induced ferromagnetic ordering of surface Ce^+3^ clustered together with Y^+3^ ions. Hence, Ce^+3^ ions stabilized at the surface replace some Y^+3^ ions in the M^+3^-V_O_ clusters already observed in bulk. Similarly, Sm^3+^-V_O_-Ce^3+^ complexes were observed for *x* > 0.07 on the surface of Sm-doped nanoparticles (Chen et al., 2014), suggesting a core-shell defect structure, already proposed in Malecka et al. (2008); Małecka et al. (2009) in terms of surface RE segregation.

Recently, 3D electron microscopy revealed the 3D surface of La-doped ceria with subnanometric resolution (Collins et al., 2017). Additional V_O_s were seen within the first 1.5 nm of the particle surface together with associated changes in Ce(4f) hybridization as well as surface enrichment in La. Acharya et al. (2014) observed through Raman and EXAFS that in nanoparticles Gd induces more intrinsic V_O_s than Sm. For a fixed doping amount, reducing the particle size damped the Raman signal at 370 cm^−1^, which is the fingerprint of C-type ordering (6-fold coordination). Accordingly, the C-type domains observed in bulk samples disappear, or reduce in intensity, when decreasing particle size. No PDF peak of C-ordering is observed for *x* = 0.313 Gd-doped samples (Coduri et al., 2017) when particle size is below 10–15 nm. Similarly, lower sintering temperatures in Y-doped samples (*x* = 0.10 and 0.25) led to smaller nanodomains (Ou et al., 2006a). Eventually, Sen et al. (2008) observed that nanometric particle size doubles the population of Y(VIII), increasing the probability of V_O_s to be NN as Ce, which can thus be reduced to +3. This can enhance the migration of V_O_s, which are less strongly bound to Ce^+3^ than to Y^+3^ (Mogensen et al., 2000).

In conclusion, reducing particle size to the nanometric scale increases the fraction of Ce^+3^, mostly located at the surface and strongly associated with V_O_s. Ce^+3^ acts as a dopant even larger than Nd^+3^ and La^+3^, which are known to shift the long range F to C phase transformation to higher dopant concentrations. Ce^3+^ thus stabilizes fluorite. XRD, PDF and Raman provided evidence in this direction.

### From low temperature to operating conditions

The above investigations were performed at low temperature, which is the best condition for structural analysis. Yet, this is far from real operating conditions, i.e., high temperature and controlled (oxidizing and reducing) atmosphere. Only a few investigations reported in the literature were not performed at ambient condition, and generally they are carried out under air. This is due firstly to instrumental difficulties, such as the case of electron microscopy. Secondly, increasing atomic vibrations broaden the signals of local probes, undermining the significance of local scale investigations. High temperature does not affect diffraction studies, other than making intensity decaying faster with momentum transfer. Since doped ceria retains its fluorite structure on a wide temperature range, no structural discontinuity has ever been reported. Yashima used high temperature powder diffraction to probe static disorder by analyzing APDs (Yashima and Takizawa, 2010). No deviation from linearity with temperature of lattice parameters and APDs using neutron diffraction on x(La) = 0.25 up to 800°C (Coduri et al., 2013b) was observed, nor it was for different compositions of Sm (Artini et al., 2018) and Gd (Artini et al., 2014). This evidences once more the need for a local probe. To the authors' knowledge, only Wang et al. (2006) used EXAFS on x(Y) = 0.10 doped ceria at high temperature (600°C). Although Y- and Ce-environments appeared more homogeneous at high temperature, they claimed that structural features become ambiguous because of excessive signal broadening.

Raman suffers as well from high temperature effects. Still, the Raman band assigned to dopant-V_O_ clusters was found to vanish at 450°C for x(Gd) = 0.15, consistently with the dissociation of V_O_s from clusters involving dopant ions. These clusters are preserved for higher dopant amount. A similar behavior was found for other dopants (Shirbhate et al., 2016). PDF showed that local C-type orderings are retained up to 750°C, even though to a lesser extent than at RT. Further distortions, compatible with Ce reduction, were observed under reducing atmosphere (Coduri et al., 2013b).

HRTEM was used to compare materials before and after being subject to operating conditions, revealing that reducing atmosphere induces further V_O_s ordering consequent to Ce reduction (Li et al., 2013). Increasing sintering temperature promotes a melting, at least a partial one, of the clusters/domains rich in V_O_s. Surprisingly, among the vast set of reports in the literature, very little effort has been dedicated so far to move defect structures investigations toward real operating conditions.

## Atomistic modeling methods of doped ceria

### AB initio calculations

Density Functional Theory (DFT) is currently the computational workhorse for the first principles modeling of doped ceria, surfaces and catalytic reactivity. However, solving the Schrödinger equation with standard DFT methods presents one problem, that is, the spurious self-interaction between electrons. The substitution of Ce^4+^ with a trivalent ion leaves an unpaired hole in the system. It turns out that in many oxides, such as MgO, TiO_2_, SiO_2_, CeO_2_, both local (LDA) and semilocal (GGA: BLYP, PW91, PBE, PBEsol) functionals yield a hole wavefunction which is delocalized on several ionic sites. Indeed, reduced CeO_2_ is predicted to be metallic by GGA functionals, in stark contrast with experiments. Moreover, EPR experiments, through the hyperfine couplings, reveal that the spin-density is very much localized and UPS experiments show that new states, associated with Ce *4f* orbitals, appear at ~1.2 eV above the top of the valence band. This drawback carries several additional consequences and leads to wrong predictions about lattice spacing, vacancy formation and migration energies, surface energies, catalytic activity (Pacchioni, 2008).

Two practical approaches emerged to partially correct for the self-interaction issue: DFT+Hubbard U (Dudarev et al., 1998; Cococcioni and de Gironcoli, 2005) and hybrid functionals (Dovesi et al., 2005, 2014). Both methods are rooted in the principle that Hartree-Fock (HF) is almost free of self-interaction. In fact, DFT+U can be viewed as a local-HF correction, acting on the subspace of localized *d* and *f* electrons. Similarly, hybrid functionals evaluate the exchange potential by mixing pure orbital-dependent HF and density-based exchange. Hybrid functionals like B3LYP and PBE0 retain the long range Coulomb interaction while the HSE (Heyd et al., 2003) uses a screened Coulomb interaction, which appears to be more appropriate to deal with periodic solids. However, both methods depend on empirical parameters: the value of U and the fraction of HF, respectively, which makes them not fully *ab-initio*.

**Figure 9:**
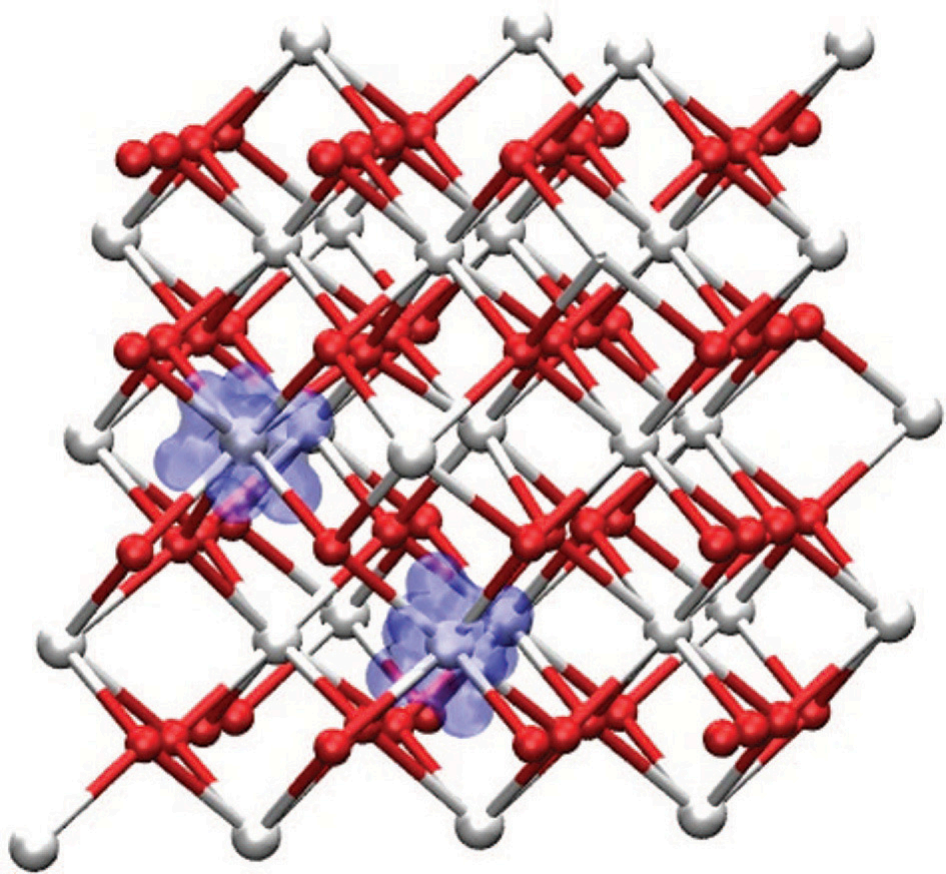
Spin density of bulk CeO_2_ in presence of an oxygen vacancy, showing the formation of two Ce^3+^ centers localized around the vacancy. Reprinted with permission from Plata et al. (2012). © 2012 American Institute of Physics.

There are procedures to determine the *U*-values (Cococcioni and de Gironcoli, 2005) and the fraction of HF from the dielectric constant (Skone et al., 2014) but they are not widespread in calculations. Rather, it is common practice to adjust these parameters to reproduce some experimental quantities, such as lattice spacing or vacancy formation energy (Fabris et al., 2005a; Pacchioni, 2008). Lu and Liu (2014), presented a rationalization of the Hubbard U parameter for Ce oxides, calculated within different approaches (linear response, constrained RPA). They found that the U parameter ranges from ~4.3 to 6.7 eV and depends strongly on the Ce-O coordination number and bond length. A similar conclusion was reported in Loschen et al. (2007). Recently it has been found that adding a Hubbard U term on oxygen *2p* orbital (despite the fact that is not as localized like the *4f* orbitals), does improve the electronic structure and the energetics of defective ceria (Yeriskin and Nolan, 2010; Plata et al., 2012). In particular, Figure 9 shows the spin density around a V_O_. By employing DFT+U spin density remains strictly localized on two Ce^3+^ ions. Without U corrections, it would spread over many more sites.

Hybrid functionals constitute a valid alternative to DFT+U, at the price of a higher computational cost, especially in the plane-waves. Among all hybrid functionals, the screened-exchange HSE06 functional is the most used to describe bulk CeO_2_ and Ce_2_O_3_ (Hay et al., 2006; Da Silva et al., 2007; Ganduglia-Pirovano et al., 2007; Du et al., 2018), oxygen vacancies at surfaces (Nolan, 2010, 2011; Han et al., 2016), polarons (Sun et al., 2017) and dopants (Shi et al., 2016). Other non-screened functionals, such as B3LYP and B3PW91 were employed successfully to calculate the optical properties of bulk CeO_2_ (El Khalifi et al., 2016). In general, hybrid functionals provide a better description of both Ce^3+^ and Ce^4+^ ions, larger band gaps, larger vacancy formation energies and more localized hole wavefunctions, with respect to pure DFT and DFT+U. Finally, there have been only few but promising investigations on bulk CeO_2_ employing meta-GGA functionals (Tran et al., 2006).

Most calculations reported in literature employ the plane-wave pseudopotential method (Kresse and Joubert, 1999; Giannozzi et al., 2009) and all-electron local-basis calculations (Dovesi et al.) are less frequent due to the large number of electrons of the lanthanides. Pseudopotentials instead treat explicitly only valence electrons and they often discard the *4f* electrons (putting them in the core) in order to reduce the computational cost and complexity (i.e., discarding magnetism; Dholabhai et al., 2010).

To describe different doping concentrations, one typically builds periodic supercells consisting of 96 sites (2 × 2 × 2 of the conventional cubic CeO_2_). In the presence of multiple vacancies, one must pay attention to spin multiplicity and to the fact that DFT+U can present multiple local minima depending on the starting guess of the orbital occupations. It was found that the energy difference between parallel and anti-parallel spins is very small (hence, a ferromagnetic solution can be imposed, Murgida et al., 2014) and that the full randomization of the starting guess can confidently provide the true electronic ground state.

To determine the optimal dopants and vacancies configuration is a formidable task even for a 96-atoms supercell, where the number of combinations can be very large. To solve this problem, genetic optimization algorithms proved to be efficient (Jung et al., 2018). However, such periodic supercells are too small to calculate accurately the thermodynamics, vacancy ordering and clustering effects. To overcome the finite-size effects, several authors employed *cluster expansion* techniques (van de Walle et al., 2002). In short, the idea is to map the local atomic configurations of dopants and vacancies on a Heisenberg lattice model. The “J” parameters of Heisenberg Hamiltionian are obtained by total energy differences between different configurations. Next, Monte Carlo methods are used to include temperature effects and to provide useful insights on vacancy order/disorder and on the microscopic structure at the nanometer scale (Gopal and Van De Walle, 2010; Murgida et al., 2014; Žguns et al., 2017, 2018).

Despite the tremendous progress of first-principles techniques and code in the past few years, both DFT+U and hybrid functionals still need to be improved in their formulation or in their parametrization. This would reduce the dependence on experimental inputs and provide more accurate, *ab-initio* predictions. The development of DFT-based tight-binding methods (i.e., DFTB) allows to simulate larger supercells that can represent closely the local structure, the clustering and ordering of defects, minimizing the spurious interaction between their periodic replicas (Kullgren et al., 2017).

### Interatomic potentials

As shown above, the combination of DFT methods and cluster expansion techniques is extremely powerful but also computationally involved. In addition to first-principle methods, doped ceria have also been studied by Molecular Dynamics (MD) and Monte Carlo (MC) methods, using empirical potentials. The most common potentials are pairwise Born and Mayer (1932) rigid-ion potentials. In particular Vives and Meunier (2015) studied the defect structure and defect migration paths with six different parametrizations of the BM potentials. By comparing to experimental data they found that only two parameter sets can be used confidently to study Gd-doped CeO_2_. For a recent review of atomistic simulations of oxide interfaces, surfaces and nanoparticles (see Sayle and Sayle, 2007).

### Calculated properties

In this section we selected from the vast literature recent results on ab-initio modeling of doped ceria. Vanpoucke et al. (2014) studied extensively the aliovalent doping of CeO_2_ with lanthanide, transition metal, and alkali atoms with DFT+U and determined the parameters of the Vegard's law, the defect formation energy, bulk modulii, thermal expansion parameters as a function of dopant concentration. Apostolov et al. (2018) and Jung et al. (2018) report phonon calculations and Raman shifts of the *F*_2*g*_ mode of pure CeO_2_, as a function of doping. In particular, doping with lanthanide +*3* ions (whose Shannon radius is larger than that of Ce^4+^) causes a decrease in the frequency of the *F*_2*g*_ Raman mode and considerable local structure relaxations. Dholabhai et al. (2010) and Ahn et al. (2012) and their coworkers studied the oxygen vacancy migration barriers in Pr-doped ceria. Surfaces of pure and doped ceria have been studied in Yeriskin and Nolan (2010) and Farra et al. (2013) where they have found the appearance of Ce^3+^ and one compensated oxygen vacancy. The electronic structure of planar and stepped surface was studied by Fabris et al. (2005b) and Esch et al. (2005).

### Ionic conductivity

The ionic conductivity of doped and co-doped ceria has been studied by MD with empirical potential by Burbano et al. (2014) using a sophisticated polarizable ion model fitted to ab-initio results. Their main finding is that oxygen conductivity in co-doped ceria can be well approximated by the weighted average of the conductivity of the single-doped parent compounds. According to their analysis the oxygen conductivity is determined by the local lattice strain generated by the single defect rather than by synergistic effects between different dopants. This confirms EXAFS investigations (Yoshida et al., 2001).

Purton et al. (2017) simulated the ionic conductivity of Ca- and Gd-doped CeO_2_ using large simulation cells. They employed a hybrid-MC technique consisting of a short (1 fs) MD run followed by a MC exchange move between a Ce and a dopant ion. The purpose of the MC move is to provide an escape path from local-minima configurations in order to approach the equilibrium in a faster way. They found that for x(Gd)>0.2, Gd-rich domains are formed. They also calculated the oxygen conductivity for randomized Gd positions and Gd-nanodomains. They found, in accordance with percolation theory, that Gd-rich domains limit oxygen mobility. They also studied the segregation of the dopants at grain boundaries of CeO_2_.

Koettgen et al. (2018) reported an ab-initio study of the oxygen conductivity, employing DFT and Kinetic Monte Carlo (KMC). As already mentioned above, they considered only jumps in the <100> direction by half of the unit cell, and only three local configurations (Ce-Ce, Ce-RE, RE-RE). Despite the small database of moves, their KMC results are in very good agreement with experiments. Contrary to observations of Burbano et al. (2014), they found that the ionic conductivity is influenced by trapping, blocking and vacancy–vacancy synergistic interactions: blocking limits the dopant fraction at the ionic conductivity maximum while trapping limits the maximum ionic conductivity. They also found a non-linear Arrhenius behavior of the conductivity, with a reduced activation energy at high temperature, which they ascribe to the “association” between an oxygen ion and a rare-earth dopant.

### Local structure

The thermodynamics of defective ceria was studied by cluster expansion methods by Gopal and Van De Walle (2010); Žguns et al. (2017, 2018). In particular, Gopal et al. addressed the thermodynamics of intrinsic oxygen vacancies. In addition to configurational entropy they also included the lattice vibrational contribution to the free energy. Their lattice Monte Carlo simulations showed that vacancies have a tendency to cluster and to order along preferred directions. DFT calculations showed for instance that for small radius dopants (La to Nd), the oxygen vacancy tend to occupy the nearest-neighbor (NN) position, whereas large radius dopants (Sm to Er) the oxygen vacancy occupies the next-nearest-neighbor (NNN) site, in order to minimize the elastic energy (Andersson et al., 2006; Gupta et al., 2010). This is good agreement with EXAFS experiments (see Section X-Ray Absorption Spectroscopy). Using empirical potentials, Hayashi et al. (2000) showed that by doping with La, Gd, and Y, the local structure of the dopant-vacancy complex is characterized by oxygen relaxation along the [100] direction. As a consequence the Ce-O distance decreases even if the average volume of the fluorite cell is increased.

The ordering of Gd dopants was studied extensively by Žguns and coworkers in two recent papers (Žguns et al., 2017, 2018). Their Monte Carlo simulations of both thermal equilibrium and rapid quenching showed a critical temperature just below 1,000 K. Their simulation showed that below *T*_c_ Gd tends to phase-separate from CeO_2_, forming nano-domains of C-type Gd_2_O_3_ (see Figure 8 of Žguns et al., 2017). At high temperature the Gd ions take a completely random distribution but oxygen vacancies tend to cluster in the coordination shell of the dopant. They further studied the entire CeO_2_-Gd_2_O_3_ phase diagram, in the full concentration range, as a function of temperature (Žguns et al., 2018).

## Concluding remarks

Ce_1−x_RE_x_O_2−x/2_ systems are very simple in appearance. Only three elements are involved in each solid solution among which RE in most cases exploit one oxidation state (+3) thus reducing the possible point defects to *RE*′_Ce_, $V_{O}^{••}$ and electrons in the conduction band. Their crystallographic structure is even simpler: in F solid solution cations and oxygen ions occupy one special positions each; even structural transitions to the C-type structure add only few positional degrees of freedom. Nevertheless, they show complex transport properties in dependence to RE nature and concentration.

In some sense, their behavior resembles the chess endgame when a small number of pieces generate a gigantic number of possible positions and move sequences (Matanović, 2012). Likewise, the few chemical elements involved in Ce_1−x_RE_x_O_2−x/2_ phases generate extremely complex defect architectures.

In this paper, we have reviewed some recent conductivity, diffraction, spectroscopy, microscopy and computation results on Ce_1−x_RE_x_O_2−x/2_ materials, underlying the interplay of different techniques. In particular, an accurate structural characterization at different scale length is found to be fundamental to rationalize their physical properties.

## Author contributions

MC wrote the most part of section Experimental Structural Probes and contributed to the general organization of the review paper. SC wrote a part of section Experimental Structural Probes. ML wrote section Technological Applications of CeO_2_-based materials. DC wrote section Atomistic modeling methods of doped ceria. MS wrote sections Introduction, Defect Chemistry and Transport Properties, Concluding remarks and coordinated the author contributions.

### Conflict of interest statement

The authors declare that the research was conducted in the absence of any commercial or financial relationships that could be construed as a potential conflict of interest. The handling editor declared a past co-authorship with several of the authors (MC and MS).

## References

[B1] Acharya S. A. Gaikwad V. M. D'Souza S. W. Barman S. R. (2014). Gd/Sm dopant-modified oxidation state and defect generation in nano-ceria. Solid State Ionics 260, 21–29. 10.1016/j.ssi.2014.03.008

[B2] Adachi G. Y. Imanaka N. (1998). The binary rare earth oxides. Chem. Rev. 98, 1479–1514. 10.1021/cr940055h 11848940

[B3] Adijanto L. Sampath A. Yu A. S. Cargnello M. Fornasiero P. Gorte R. J. (2013). Synthesis and stability of Pd@CeO_2_ core–shell catalyst films in solid oxide fuel cell anodes. ACS Catal. 3, 1801–1809. 10.1021/cs4004112

[B4] Ahn K. Yoo D. S. Prasad D. H. Lee H. W. Chung Y. C. Lee J. H. (2012). Role of multivalent Pr in the formation and migration of oxygen vacancy in Pr-doped ceria: experimental and first-principles investigations. Chem. Mater. 24, 4261–4267. 10.1021/cm3022424

[B5] Ainscough J. B. Moore D. A. Osborn S. C. (1975). The kinetics of the C + B transformation in europium sesquioxide. J. Nucl. Metar. 55, 229–232. 10.1016/0022-3115(75)90156-7

[B6] Allieta M. Brunelli M. Coduri M. Scavini M. Ferrero C. (2011). Differential Pair Distribution Function applied to Ce_1−x_Gd_x_O_2−x/2_ system. Zeitschrift fu r Kristallographie Proceedings 1, 15–20. 10.1524/zkpr.2011.0002

[B7] Anderson M. P. Cox D. E. (1983). Neutron diffuse scattering in Y_2_O_3_- and Sc_2_O_3_-doped CeO_2_. Solid State Lonics 9 and 10, 953–960. 10.1016/0167-2738(83)90116-9

[B8] Andersson D. A. Simak S. I. Skorodumova N. V. Abrikosov I. A. Johansson B. (2006). Optimization of ionic conductivity in doped ceria. P. Natl. Acad. Sci. U.S.A. 103, 3518–3521. 10.1073/pnas.0509537103 16478802 PMC1450116

[B9] Andrievskaya E. R. Kornienko O. A. Sameljuk A. V. Sayir A. (2011). Phase relation studies in the CeO_2_-La_2_O_3_ system at 1100-1500 C. J. Eur. Ceram. Soc. 31, 1277–1283. 10.1016/j.jeurceramsoc.2010.05.024

[B10] Anselmi-Tamburini U. Maglia F. Chiodelli G. Tacca A. Spinolo G. Riello P. (2006). Nanoscale effects on the ionic conductivity of highly doped bulk nanometric Cerium Oxide. Adv. Funct. Mater. 16, 2363–2368. 10.1002/adfm.200500415

[B11] Apostolov A. T. Apostolova I. N. Wesselinowa J. M. (2018). Theoretical study of the phonon properties of pure and ion doped CeO_2_ nanoparticles. Sol. State Comm. 279, 17–21. 10.1016/j.ssc.2018.05.007

[B12] Argyriou D. N. (1994). Measurement of the static disorder contribution to the temperature factor in cubic stabilized ZrO2. J. Appl. Crystallogr. 27, 155–158. 10.1107/S0021889893007964

[B13] Artini C. Carnasciali M. M. Viviani M. Presto S. Plaisier J. R. Costa G. A. (2018). Structural properties of Sm-doped ceria electrolytes at the fuel cell operating temperatures. Solid State Ionics 315, 85–91. 10.1016/j.ssi.2017.12.009

[B14] Artini C. Carnascialia M. M. Plaisier J. R. Costa G. A. Pani M. (2017). A novel method for the evaluation of the Rare Earth (RE) coordination number in RE-doped ceria through Raman spectroscopy. Solid State Ionics 311, 90–97. 10.1016/j.ssi.2017.09.016

[B15] Artini C. Costa G. A. Pani M. Lausi A. Plaisier J. (2012). Structural characterization of the CeO_2_/Gd_2_O_3_ mixed system by synchrotron X-ray diffraction. J. Sol. State Chem. 190, 24–28. 10.1016/j.jssc.2012.01.056

[B16] Artini C. Pani M. Carnasciali M. M. Buscaglia M. T. Plaisier J. R. Costa G. A. (2015). Structural features of Sm- and Gd-doped ceria studied by synchrotron X-ray diffraction and μ-raman spectroscopy. Inorg. Chem. 54, 4126–4137. 10.1021/acs.inorgchem.5b00395 25849073

[B17] Artini C. Pani M. Carnasciali M. M. Plaisier J. R. Costa G. A. (2016). Lu-, S m-, and Gd-doped Ceria: a comparative approach to their structural properties. Inorg. Chem. 55, 10567–10579. 10.1021/acs.inorgchem.6b01806 27681325

[B18] Artini C. Pani M. Lausi A. Masini R. Costa G. A. (2014). High temperature structural study of Gd-soped ceria by synchrotron X-ray diffraction (673 K ≤ T ≤ 1073 K). Inorg. Chem. 53, 10140–10149 10.1021/ic5011242 25192043

[B19] Balazs G. B. Glass R. S. (1995). ac impedance studies of rare earth oxide doped ceria. Solid State Ionics 76, 155–162. 10.1016/0167-2738(94)00242-K

[B20] Banerji A. Grover V. Sathe V. Deb S. K. Tyagi A. K. (2009). system: unraveling of microscopic features by raman spectroscopy. Solid State Comm. 149, 1689–1692. 10.1016/j.ssc.2009.06.045

[B21] Bellière V. Joorst G. Stephan O. de Groot F. M. Weckhuysen B. M. (2006). Phase segregation in Cerium -Lanthanum solid solutions. J. Phys. Chem. B, 110, 9984–9990. 10.1021/jp060882+ 16706456

[B22] Bertolotti F. Dirin D. N. Ibáñez M. Krumeich F. Cervellino A. Frison R.. (2016). Crystal symmetry breaking and vacancies in colloidal lead chalcogenide quantum dots. Nat. Mater. 15, 987–994. 10.1038/nmat4661 27295101

[B23] Bevan D. J. M. Kordis J. (1964). Mixed oxides of the type MO_2_ (fluorite)-M_2_O_3_ -I oxygen dissociation pressures and phase relationships in the system CeO_2_-Ce_2_O_3_ at high temperatures. J. Inorg. Nucl. Chem. 26, 1509–1523. 10.1016/0022-1902(64)80038-5

[B24] Bevan D. J. M. Summerville E. (1979). Mixed rare earth oxides, in Handbook on the Physics and Chemistry of Rare Earths, ed. Gschneidner K. A. Jr. Eyring L. (Oxford: North-Holland Publishing Company), 423.

[B25] Born M. Mayer J. E. (1932). Zur gittertheorie der ionenkristalle. Z. Phys 75, 1–18. 10.1007/BF01340511

[B26] Burbano M. Nadin S. Marrocchelli D. Salanne M. Watson G. (2014) Ceria co-doping: synergistic or average effect? Phys. Chem. Chem. Phys. 16, 8320–8331. 10.1039/c4cp00856a 24658460

[B27] Cervellino A. Frison R. Bertolotti F. Guagliardi A. (2015). DEBUSSY2.0: the new release of a Debye user system for nanocrystalline and/or disordered materials. J. Appl. Cryst. 48, 2026–2032. 10.1107/S1600576715020488

[B28] Chavan S. V. Mathews M. D. Tyagi A. K. (2004). Phase relations and thermal expansion studies in the Ceria-Yttria system. J. Am. Ceram. Soc. 87, 1977–1980. 10.1111/j.1151-2916.2004.tb06349.x

[B29] Chavan S. V. Mathews M. D. Tyagi A. K. (2005). Phase relations and thermal expansion studies in the CeO_2_-NdO_1.5_ system. Mater. Res. Bull. 40, 1558–1568. 10.1016/j.materresbull.2005.04.014

[B30] Chavan S. V. Tyagi A. K. (2005). Phase relations and lattice thermal expansion studies in the Ce_0.50_RE_0.5_0O_1.75_ (RE = rare-earths). Mater. Sci. Eng. A 404, 57–63. 10.1016/j.msea.2005.05.036

[B31] Checchia S. Scavini M. Allieta M. Brunelli M. Ferrero C. Coduri M. (2015). Size and spatial correlation of defective domains in yttrium-doped CeO_2_. Powder Diffr. 30, S119–S126. 10.1017/S0885715615000135

[B32] Chen S. Y. Chen R. J. Lee W. Dong C. L. Gloter A. (2014). Spectromicroscopic evidence of interstitial and substitutional dopants in association with oxygen vacancies in Sm-doped ceria nanoparticles. Phys. Chem. Chem. Phys. 16:274. 10.1039/c3cp54613f 24413060

[B33] Chen T. P. Wright J. D. Krist K. Singhal S. C. Tagawa H. Lehnert W. (Eds.) (1997). SOFC V (The Electrochemical Society), 69.

[B34] Chen W. Navrotsky A. (2006). Thermochemical study of trivalent-doped ceria systems: CeO_2_-MO_1.5_ (M = La, Gd, and Y). J. Mater. Res. 21, 3242–3251. 10.1557/jmr.2006.0400

[B35] Chiang Y.-M. Lavik E. B. Kosacki I. Tuller H. L. Ying J. Y. (1996). Defect and transport properties of nanocrystalline CeO_2−x_. Appl. Phys. Lett. 69, 185–187. 10.1063/1.117366

[B36] Chueh W. C. Hao Y. Jung W. Haile S. M. (2011). High electrochemical activity of the oxide phase in model ceria–Pt and ceria–Ni composite anodes. Nat. Mater. 11, 155–161. 10.1038/nmat3184 22138788

[B37] Cococcioni M. de Gironcoli S. (2005). Linear response approach to the calculation of the effective interaction parameters in the LDA+ U method. Phys. Rev. B, 71:035105 10.1103/PhysRevB.71.035105

[B38] Coduri M. (2013). PhD thesis: “Local Disorder in doped ceria: a crystallographic study.” Available online at: https://air.unimi.it/retrieve/handle/2434/215536/261641/phd_unimi_R08772.pdf

[B39] Coduri M. Brunelli M. Scavini M. Allieta M. Masala P. Capogna L. (2012a). Rare Earth doped ceria: a combined X-ray and neutron pair distribution function study. Z. Kristallogr. 227, 272–279. 10.1524/zkri.2012.1493

[B40] Coduri M. Masala P. Allieta M. Peral I. Brunelli M. Biffi C. A.. (2018). Phase Transformations in the CeO_2_ – Sm_2_O_3_ System: a multiscale powder diffraction investigation. Inorg. Chem. 57, 879–891. 10.1021/acs.inorgchem.7b02896 29280608

[B41] Coduri M. Scavini M. Allieta M. Brunelli M. Ferrero C. (2012b). Local disorder in yttrium doped ceria (Ce_1−x_Y_x_O_2−x/2_) probed by joint X-ray and Neutron Powder Diffraction. J. Phys. 340:012056 10.1088/1742-6596/340/1/012056

[B42] Coduri M. Scavini M. Allieta M. Brunelli M. Ferrero C. (2013a). Defect structure of Y-doped ceria on different length scales. Chem. Mater. 25, 4278–4289. 10.1021/cm402359d

[B43] Coduri M. Scavini M. Brunelli M. Masala P. (2013b). In situ pair distribution function study on lanthanum doped ceria. Phys. Chem. Chem. Phys. 15, 8495–8505. 10.1039/c3cp44300k 23403753

[B44] Coduri M. Scavini M. Brunelli M. Pedrazzin E. Masala P. (2014). Structural characterization of Tb- and Pr-doped ceria. Solid State Ionics 268, 150–155. 10.1016/j.ssi.2014.10.020

[B45] Coduri M. Scavini M. Pani M. Carnasciali M. M. Klein H. Artini C. (2017). From nano to microcrystals: effects of different synthetic pathways on the defect architecture in heavily Gd-doped ceria. Phys. Chem. Chem. Phys. 19, 11612–11630. 10.1039/C6CP08173H 28428993

[B46] Collins S. M. Fernandez-Garcia S. Calvino J. J. Midgley P. A. (2017). Sub-nanometer surface chemistry and orbital hybridization in lanthanum-doped ceria nanocatalysts revealed by 3D electron microscopy. Sci. Rep. 7:5406 10.1038/s41598-017-05671-9 28710378 PMC5511253

[B47] Da Silva J. L. F. Ganduglia-Pirovano M. V. Sauer J. Bayer V. Kresse G. (2007). Hybrid functionals applied to rare-earth oxides: the example of ceria. Phys. Rev. B 57:045121 10.1103/PhysRevB.75.045121

[B48] DeCaluwe S. C. Grass M. E. Zhang C. El Gabaly F. Bluhm H. Liu Z. (2010). In situ characterization of ceria oxidation states in high-temperature electrochemical cells with ambient pressure XPS. J. Phys. Chem. C. 114, 19853–19861. 10.1021/jp107694z

[B49] Deguchi H. Yoshida H. Inagaki T. Horiuchi M. E. X. A. F. S. (2005). Study of doped ceria using multiple data set fit. Solid State Ionics 176, 1817–1825. 10.1016/j.ssi.2005.04.043

[B50] Dholabhai P. P. Adams J. B. Crozier P. Sharma R. (2010). Oxygen vacancy migration in ceria and Pr-doped ceria: a DFT+U study. J. Chem. Phys. 132:094104. 10.1063/1.3327684 20210386

[B51] Dikmen S. Shuk P. Greenblatt M. (1999). Hydrothermal synthesis and properties of Ce_1−x_La_x_O_2−delta_ solid solutions. Solid State Ionics 126, 89–95. 10.1016/S0167-2738(99)00146-0

[B52] Dohčević-Mitrović Z. D. Šćepanović M. J. Grujić-Brojčin M. U. Popović Z. V. Bošković S. B. Matović B. M. (2006). The size and strain effects on the Raman spectra of Ce1–xNdxO2–δ (0 ≤ x ≤ 0.25) nanopowders. Solid State Comm. 137, 387–390. 10.1016/j.ssc.2005.12.006

[B53] Dovesi R. Orlando R. Civalleri B. Roetti C. Saunders V. R. Zicovich-Wilson C. M. Z. (2005). CRYSTAL: a computational tool for the *ab initio* study of the electronic properties of crystals. Kristallography 220, 571–573. 10.1524/zkri.220.5.571.65065

[B54] Dovesi R. Orlando R. Erba A. Zicovich-Wilson C. M. Civalleri B. Casassa S. (2014). CRYSTAL14: A program for the Ab initio investigation of crystalline solids. Int. J. Quantum Chem. 114, 1287–1317. 10.1002/qua.24658

[B55] Du D. Wolf M. J. Hermansson K. Broqvist P. (2018). Screened hybrid functionals applied to ceria: effect of fock exchange. Phys. Rev. B 97:235203 10.1103/PhysRevB.97.235203

[B56] Duboviks V. Lomberg M. Maher R. C. Cohen L. F. Brandon N. P. Offer G. J. (2015). Carbon deposition behaviour in metal-infiltrated gadolinia doped ceria electrodes for simulated biogas upgrading in solid oxide electrolysis cells. J. Power Sourc. 293, 912–921. 10.1016/j.jpowsour.2015.06.003

[B57] Duboviks V. Maher R. Kishimoto M. Cohen L. Brandon N. Offer G. (2014). A Raman spectroscopic study of the carbon deposition mechanism on Ni/CGO electrodes during CO/CO_2_ electrolysis. Phys. Chem. Chem. Phys. 16, 13063–13068. 10.1039/C4CP01503G 24871047

[B58] Dudarev S. L. Botton G. A. Savrasov S. Y. Humphreys C. J. Sutton A. P. (1998). Electron-energy-loss spectra and the structural stability of nickel oxide: An LSDA+U study. Phys. Rev. B 57, 1505–1509. 10.1103/PhysRevB.57.1505

[B59] Egami T. Billinge S. J. L. (2003). Underneath Bragg Peaks. Oxford, UK; Amsterdam; San Diego, CA: Elsevier Ltd.

[B60] Eguchi K. Setoguchi T. Inoue T. Arai H. (1992). Electrical properties of ceria-based oxides and their application to solid oxide fuel cells. Solid State Ionics 52, 165–172. 10.1016/0167-2738(92)90102-U

[B61] El Khalifi M. Picaud F. Bizi M. (2016). Electronic and optical properties of CeO2 from first principles calculations. Anal. Methods 8, 5045–5052. 10.1039/C6AY00374E

[B62] Esch F. Fabris S. Zhou L. Montini M. Africh C. Fornasiero P.. (2005). Electron localization determines defect formation on ceria substrates. Science 309, 752–755. 10.1126/science.1111568 16051791

[B63] Faber J. Geoffroy C. Roux A. Sylvestre A. Abelard P. (1989). A Systematic Investigation of the dc Electrical Conductivity of Rare-Earth Doped Ceria. Appl. Phys. A 49, 225–232 10.1007/BF00616848

[B64] Fabris S. de Gironcoli S. Baroni S. Vicario G. Balducci G. (2005a). Taming multiple valency with Density Functional: the case of defective ceria. Phys. Rev. B. 71:041102 10.1103/PhysRevB.71.041102

[B65] Fabris S. Vicario G. Balducci G. de Gironcoli S. Baroni S. (2005b). Electronic and atomistic structure of clean and reduced ceria surfaces. J. Phys. Chem. B, 109, 22860–22867. 10.1021/jp0511698 16853978

[B66] Farra R. Garcia-Melchor M. Eichelbaum M. Hashagen M. Frandsen W. Allan J. (2013). Promoted ceria: a structural, catalytic, and computational study. ACS Catalysis 3, 2256–2268. 10.1021/cs4005002

[B67] Feng Z. A. El Gabaly F. Ye X. Shen Z. X. Chueh W. C. (2014). Fast vacancy-mediated oxygen ion incorporation across the ceria-gas electrochemical interface. Nat. Commun. 5:4374. 10.1038/ncomms5374 25007038

[B68] Ganduglia-Pirovano M. V. Hoffmann A. Sauer J. (2007). Oxygen vacancies in transition metal and rare earth oxides: current state of understanding and remaining challenges. Surf. Sci. Rep. 62, 219–270. 10.1016/j.surfrep.2007.03.002

[B69] Gateshki M. Niederberger M. Deshpande A. S. Ren Y. Petkov V. (2007). Atomic-scale structure of nanocrystalline CeO_2_ –ZrO_2_ oxides by total x-ray diffraction and pair distribution function analysis. J. Phys. Condens. Matter 19:156205 10.1088/0953-8984/19/15/156205

[B70] Giannici F. Gregori G. Aliotta C. Lono A. Maier J. Martorana A. (2014). Structure and oxide-ion conductivity: local order, defect interactions and grain boundary effects in acceptor-doped ceria. Chem. Mater. 26, 5594–6006. 10.1021/cm502810e

[B71] Giannozzi P. Baroni S. Bonini N. Calandra M. Car R. Cavazzoni C.. (2009). Quantum Espresso: a modular and open-source software project for quantum simulations of materials. J. Phys. 21:395502. 10.1088/0953-8984/21/39/395502 21832390

[B72] Goodenough J. (2003). Oxide-ion electrolytes. Ann. Rev. of Material Res. 33, 91–128. 10.1146/annurev.matsci.33.022802.091651

[B73] Gopal C. B. Van De Walle A. (2010). Ab initio thermo dynamics of intrinsic oxygen vacancies in ceria. Phys. Rev. B 86:134117 10.1103/PhysRevB.86.134117

[B74] Grover V. Banerji A. Sengupta P. Tyagi A. K. (2008). Raman, XRD and microscopic investigations on CeO_2_- Lu_2_O_3_ and CeO_2_- Sc_2_O_3_ systems: A sub-solidus phase evolution study. J. Solid State Chem. 181, 1930–1935. 10.1016/j.jssc.2008.04.001

[B75] Grover V. Tyagi A. K. (2004). Phase relations, lattice thermal expansion in CeO_2_-Gd_2_O_3_ system, and stabilization of cubic gadolinia. Mater. Res. Bull. 39, 859–866. 10.1016/j.materresbull.2004.01.007

[B76] Guo B. Harvey A. Risbud S. H. Kennedy I. M. (2006). The formation of cubic and monoclinic Y_2_O_3_ nanoparticles in a gas-phase flame process. Philos. Mag. Lett. 86, 457–467. 10.1080/09500830600871194

[B77] Guo X. Sigle W. Fleig J. Maier J. (2002). Role of space charge in the grain boundary blocking effect in doped zirconia. Solid State Ionics 154–155, 555–561. 10.1016/S0167-2738(02)00491-5

[B78] Gupta A. Waghmare U. V. Hegde M. S. (2010). Correlation of oxygen storage capacity and structural distortion in transition-metal-, noble-metal-, and rare-earth-ion-substituted CeO_2_ from first principles calculation. Chem. Mater. 22, 5184–5198. 10.1021/cm101145d

[B79] Hagiwara T. Kyo Z. Manabe A. Yamamura H. Nomura K. (2009). Formation of C-type rare earth structures in the Ce_1−x_Nd_x_O_2−δ_ system: a factor in the decrease in oxide-ion conductivity. J. Ceram. Soc. 117, 1306–1310. 10.2109/jcersj2.117.1306

[B80] Han X. Amrane N. Zhang Z. Benkraouda M. (2016). Oxygen vacancy ordering and electron localization in CeO2: hybrid functional study. J. Phys. Chem. C 120, 13325–13331. 10.1021/acs.jpcc.6b00865

[B81] Hartvigsen J. J. Elangovan S. Elwell J. Larsen D. (2017). Oxygen production from Mars atmosphere carbon dioxide using solid oxide electrolysis. ECS Trans. 78, 2953–2963. 10.1149/07801.2953ecst

[B82] Hay P. J. Martin R. L. Uddin J. Scuseria G. E. (2006). Theoretical study of and CeO_2_ and Ce_2_O_3_ using a screened hybrid density functional. J. Chem. Phys. 125:034712. 10.1063/1.2206184 16863378

[B83] Hayashi H. Sagawa R. Inaba H. Kawamura K. (2000). Molecular dynamics calculations on ceria-based solid electrolites with different radius dopants. Solid State Ionic 131, 281–290. 10.1016/S0167-2738(00)00675-5

[B84] Hays T. Hussain A. M. Huang Y.-L. McOwen D. W. Wachsman E. D. (2018). Improved sulfur tolerance of SOFCs through surface modification of anodes. ACS Appl. Energy Mater. 1, 1559–1566. 10.1021/acsaem.7b00354

[B85] He H. Gorte R. J. Vohs J. M. (2005). Highly sulfur tolerant Cu-Ceria anodes for SOFCs. Electrochem. Solid-State Lett. 8, A279–A280. 10.1149/1.1896469

[B86] Heinmaa I. Joon T. Kooskora H. Pahapill J. Subbi J. (2010). Local structure and oxygen ion dynamics in La doped ceria: ^17^O NMR study. Solid State Ionics 181, 1309–1315. 10.1016/j.ssi.2010.07.027

[B87] Heyd J. Scuseria G. E. Ernzerhof M. (2003). Hybrid functionals based on a screened Coulomb potential. J. Chem. Phys. 118, 8207–8215. 10.1063/1.1564060

[B88] Hooper J. Ismail A. Giorgi J. B. Woo T. K. (2010). Computational insights into the nature of increased ionic conductivity in concentrated samarium-doped ceria: a genetic algorithm study. Phys. Chem. Chem. Phys. 12, 12969–12972. 10.1039/c0cp00863j 20830388

[B89] Horlait D. Claparede L. Clavier N. Szenknect S. Dacheux N. Ravaux J.. (2011). Stability and Structural Evolution of ${\text{Ce}}_{1-\text{x}}^{\text{IV}}$Ln^III^_*x*_O_2−x/2_ Solid Solutions: a coupled μ-raman/XRD approach. Inorg. Chem. 50, 7150–7161. 10.1021/ic200751m 21714501

[B90] Hormes J. Pantelouris M. Balazs G. B. Rambabu B. (2000). X-ray absorption near edge structure (XANES) measurements of ceria-based solid electrolytes. Solid State Ionics 136–137, 945–954. 10.1016/S0167-2738(00)00533-6

[B91] Ikuma Y. Shimada E. Okamura N. (2005). Effect of Nd_2_O_3_ concentration on the defect structure of CeO_2_-Nd_2_O_3_ solid solution. J. Am. Ceram. Soc. 88, 419–423. 10.1111/j.1551-2916.2005.00076.x

[B92] Inaba H. Sagawa R. Hayashi H. Kawamura K. (1999). Molecular dynamics simulation of gadolinia-doped ceria. Solid State Ionics 122, 95–103 10.1016/S0167-2738(99)00036-3

[B93] Inaba H. Tagawa H. (1996). Ceria-based solid electrolytes. Solid State Ionics. 83, 1–16. 10.1016/0167-2738(95)00229-4

[B94] Jacobson A. J. (2010). Materials for Solid Oxide Fuel Cells. Chem. Mater. 22, 660–674. 10.1021/cm902640j

[B95] Jung D. H. Lee J. H. Kilic M. E. Soon A. (2018). Anisotropic vacancy-mediated phonon mode softening in Sm and Gd doped ceria. Phys. Chem. Chem. Phys. 20, 10048–10059. 10.1039/C8CP00559A 29620105

[B96] Jung G. B. Huang T. J. Chang C. L. (2002). Effect of temperature and dopant concentration on the conductivity of samaria-doped ceria electrolyte. J. Electrochem. Soc. 6, 225–230. 10.1007/s100080100238

[B97] Kilner J. A. (2008). Defects and conductivity in ceria-based oxides. Chem. Lett. 37, 1012–1015. 10.1246/cl.2008.1012

[B98] Kim D.-J. (1989). Lattice parameters, ionic conductivities, and solubility limits in fluorite-structure MO_2_ Oxide [M = Hf^4+^, Zr^4+^, Ce^4+^, Th^4+^, U^4+^] solid solutions. J. Am. Ceram. Soc., 72, 1415–1421. 10.1111/j.1151-2916.1989.tb07663.x

[B99] Kim N. Stebbins J. F. (2007). Vacancy and cation distribution in yttria-doped ceria: an 89Y and 17O MAS NMR Study. Chem. Mater. 19, 5742–5747. 10.1021/cm0715388

[B100] Koettgen J. Grieshammer S. Hein P. Grope B. O. H. Nakayama M. Martin M. (2018). Understanding the ionic conductivity maximum in doped ceria: trapping and blocking. Phys. Chem. Chem. Phys. 20, 14291–14321. 10.1039/C7CP08535D 29479588

[B101] Kossoy A. Wang Q. Korobko R. Grover V. Feldman Y. Wachtel E. (2013). Evolution of the local structure at the phase transition in CeO_2_-Gd_2_O_3_ solid solutions. Phys. Rev. B 87:054101 10.1103/PhysRevB.87.054101

[B102] Kresse G. Joubert D. (1999). From ultrasoft pseudopotentials to the projector augmented-wave method. Phys. Rev. B 59, 1758–1775. 10.1103/PhysRevB.59.1758

[B103] Kröger F. A. (1977). Defect chemistry in crystalline solids. Annu. Rev. Mater. Sci. 7, 449–475. 10.1146/annurev.ms.07.080177.002313

[B104] Kuemmerle E. A. Heger G. (1999). The Structures of C-Ce_2_O_3+d_, Ce_7_O_12_, and Ce_11_O_20_. J. Solid State Chem. 147, 485–500. 10.1006/jssc.1999.8403

[B105] Kullgren J. Wolf M. J. Hermansson K. Köhler C. Aradi B. Frauenheim T. (2017). Self-consistent-charge density-functional tight-binding (SCC-DFTB) parameters for ceria in 0D to 3D. J. Phys. Chem. C 121, 4593–4607. 10.1021/acs.jpcc.6b10557

[B106] Lee W. Chen S.-Y. Chen Y.-S. Dong C.-L. Lin H.-J. Chen C.-T. Gloter (2014). A. Defect Structure Guided Room Temperature Ferromagnetism of Y-Doped CeO_2_ Nanoparticles. J. Phys. Chem. C 118, 26359–26367. 10.1021/jp507694d

[B107] Lee Y.-H. Sumi H. Muroyama H. Matsui T. Eguchi K. (2013). Influence of Ni-Oxide anode thickness on performance stability in internal reforming of methane for solid oxide fuel cells. J. Electrochem. Soc. 160, F579–F584 10.1149/2.075306jes

[B108] Li W. Shi Y. Luo Y. Wang Y. Cai N. (2015). Carbon deposition on patterned nickel/yttria stabilized zirconia electrodes for solid oxide fuel cell/solid oxide electrolysis cell modes. J. Power Sources. 276, 26–31. 10.1016/j.jpowsour.2014.11.106

[B109] Li Z.-P. Mori T. Auchterlonie G. J. Zou J. Drennan J. (2011). Direct evidence of dopant segregation in Gd-doped ceria. Appl. Phys. Lett. 98:093104 10.1063/1.3556650

[B110] Li Z.-P. Mori T. Auchterlonie G. J. Zou J. Drennan J. (2013). Microstruct ure evolution of yttria- doped ceria in reducing atmosphere. Renew. Energ. 50, 494–497. 10.1016/j.renene.2012.07.019

[B111] Li Z.-P. Mori T. Ye F. Ou D. Auchterlonie G. J. Zou J. (2012). Cerium-reduction-induced defects clustering, ordering, and associated microstructure evolution in yttrium-doped ceria. J. Phys. Chem. C 116, 5435 −5443. 10.1021/jp211579f

[B112] Loschen C. Carrasco J. Neyman K. M. Illas F. (2007). First-principles LDA+U and GGA+U study of cerium oxides: Dependence on the effective U parameter. Phys. Rev. B 75:035115 10.1103/PhysRevB.75.035115

[B113] Lu D. Liu P. (2014). Rationalization of the Hubbard U parameter in CeO_x_ from first principles: unveiling the role of local structure in screening. J. Chem. Phys. 140:084101 10.1063/1.4865831 24588142

[B114] Malavasi L. Fisher C. A. Islam M. S. (2010). Oxide-ion and proton conducting electrolyte materials for clean energy applications: structural and mechanistic features. Chem. Soc. Rev. 39, 4370–4387. 10.1039/b915141a 20848015

[B115] Małecka M. A. Burkhardt U. Kaczorowski D. Schmidt M. P. Goran D. Kepinski L. (2009). Structure and phase stability of nanocrystalline Ce_1−x_Ln_x_O_2−x/2−d_ (Ln = Yb, Lu) in oxidizing and reducing atmosphere. J Nanopart. Res. 11, 2113–2124. 10.1007/s11051-008-9577-7 20376179 PMC2847157

[B116] Malecka M. A. Kepinski L. Maczka M. (2008). Structure and phase composit ion of nanocrystalline Ce_1−x_Lu_x_O_2−y_. J. Solid State Chem. 18, 2306–2312. 10.1016/j.jssc.2008.05.033

[B117] Mamontov E. Egami T. (2000). Structural defects in a nano-scale powder of CeO_2_ studied by pulsed neutron diffraction. J. Phys. Chem. Solids 61, 1345–1356. 10.1016/S0022-3697(00)00003-2

[B118] Mamontov E. Egami T. Brezny R. Koranne M. Tyagi S. (2000). Lattice defects and oxygen storage capacity of nanocrystalline ceria and ceria-zirconia J. Phys. Chem. B 104, 11110–11116. 10.1021/jp0023011

[B119] Mandal B. P. Grover V. Roy M. Tyagi A. K. (2007). X-ray diffraction and raman spectroscopic investigation on the phase relations in Yb_2_O_3_- and Tm_2_O_3_-Substituted CeO_2_. J. Am. Ceram. Soc. 90, 2961–2965. 10.1111/j.1551-2916.2007.01826.x

[B120] Mandal B. P. Grover V. Tyagi A. K. (2006). Phase relations, lattice thermal expansion in Ce_1−x_Eu_x_O_2−x/2_ and Ce_1−x_Sm_x_O_2−x/2_ systems and stabilization of cubic RE_2_O_3_ (RE: Eu, Sm). Mater. Sci. Eng. A 430, 120–124 10.1016/j.msea.2006.05.140

[B121] Martinez-Arias A. Hungria A. B. Fernandez-Garcia M. Iglesias-Juez A. Conesa J. C. Mather G. C. (2005). Cerium–terbium mixed oxides as potential materials for anodes in solid oxide fuel cells. J. Power Sources 151, 43–51. 10.1016/j.jpowsour.2005.02.079

[B122] Matanović A. (2012). Encyclopedia of Chess Endings. Belgrad: Chess Informants.

[B123] McBride J. R. Hass K. C. Poindexter B. D. Weber W. H. (1994). Raman and x-ray studies of Ce _1−x_ RE _x_ O _2−y_, where RE = La, Pr, Nd, Eu, Gd, and Tb. Appl. Phys. 76, 2435–2441. 10.1063/1.357593

[B124] McIntosh S. Gorte R. J. (2004). Direct hydrocarbon solid oxide fuel cells. Chem. Rev. 104, 4845–4866. 10.1021/cr020725g 15669170

[B125] Mogensen M. Sammes N. M. Tompsett G. A. (2000). Physical, chemical and electrochemical properties of pure and doped ceria. Solid State Ionics 129, 63–94. 10.1016/S0167-2738(99)00318-5

[B126] Montini T. Melchionna M. Monai M. Fornasiero P. (2016). Fundamentals and catalytic applications of CeO_2_-based materials. Chem. Rev. 116, 5987–6041. 10.1021/acs.chemrev.5b00603 27120134

[B127] Mori T. Drennan J. (2006). Influence of microstructure on oxide ionic conductivity in doped CeO_2_ electrolytes. J. Electroceram. 17, 749–757. 10.1007/s10832-006-6311-7

[B128] Mori T. Drennan J. Lee J.-H. Li J.-G. Ikegami T. (2002). Oxide ionic conductivity and microstructures of Sm- or La-doped CeO_2_-based systems. Solid State Ionics 154–155, 461–466. 10.1016/S0167-2738(02)00483-6

[B129] Mori T. Kobayashi T. Wang Y. Drennan J. Nishimura T. Li J.-G. (2005). Synthesis and characterization of nano-hetero-structured Dy Doped CeO_2_ solid electrolytes using a combination of spark plasma sintering and conventional sintering. J. Am. Ceram. Soc. 88, 1981–1984. 10.1111/j.1551-2916.2005.00260.x

[B130] Murgida G. E. Ferrari V. Ganduglia-Pirovano M. V. Llois A. M. (2014). Ordering of oxygen vacancies and excess charge localization in bulk ceria: a DFT+U study. Phys. Rev. B 90:115120 10.1103/PhysRevB.90.115120

[B131] Nakagawa T. Osuki T. Yamamoto T. A. Kitauji Y. Kano M. Katsura M.. (2001). Study on local structure around Ce and Gd atoms in CeO_2_-Gd_2_O_3_ binary system. J. Synchrotron Rad. 8, 740–742. 10.1107/S0909049500018148 11512915

[B132] Nakajima A. Yoshihara A. Ishigame M. (1994). Defect-induced Raman spectra in doped CeO 2. Phys. Rev. B 50, 13297–13307. 10.1103/PhysRevB.50.13297 9975521

[B133] Nakamura A. (2010). New defect-crystal-chemical approach to nonVegardianity and complex defect structure of fluorite-based MO_2_ – LnO _1.5_ solid solutions (M^4+^ = Ce, Th, Ln^3+^ = lanthanide) part I: Model description and lattice-parameter data analysis. Solid State Ionics 181, 1631–1653. 10.1016/j.ssi.2010.09.022

[B134] Nakamura T. Kobayashi T. Yashiro K. Kaimai A. Otake T. Sato K. (2008). Electrochemical behaviors of mixed conducting oxide anodes for solid oxide fuel cell. J. Electrochem. Soc. 155, B563–B569. 10.1149/1.2901047

[B135] Nakayama M. Martin M. (2009). First-principles study on defect chemistry and migration of oxide ions in ceria doped with rare-earth cations. Phys. Chem. Chem. Phys. 11, 3241–3249. 10.1039/b900162j 19370220

[B136] Navasa M. Frandsen H. U. Skafte T. L. Sundén B. Graves C. (2018). Localized carbon deposition in solid oxide electrolysis cells studied by multiphysics modeling. J. Power Sources 394, 102–113. 10.1016/j.jpowsour.2018.05.039

[B137] Nitani H. Nakagawa T. Yamanouchi M. Osuki T. Yuya M. Yamamoto T. A. (2004). XAFS and XRD study of ceria doped with Pr, Nd or Sm. Mater. Lett. 58, 2076–2081. 10.1016/j.matlet.2004.01.005

[B138] Nolan M. (2010). Hybrid density functional theory description of oxygen vacancies in the CeO_2_ (110) and (100) surfaces. Chem. Phys. Lett. 499, 126–130. 10.1016/j.cplett.2010.09.016

[B139] Nolan M. (2011). Enhanced oxygen vacancy formation in ceria (111) and (110) surfaces doped with divalent cations. J. Mater. Chem. 21, 9160–9168. 10.1039/c1jm11238d

[B140] Ohashi T. Yamazaki S. Tokunaga T. Arita Y. Matsui T. Harami T. (2005). EXAFS study of Ce_1−x_Gd_x_O_2−x/2_. Solid State Ionics 113–115, 559–564. 10.1016/S0167-2738(98)00322-1

[B141] Oliva C. Scavini M. Ballabio O. Sin A. Zaopo A. Dubitsky Y. (2004). Percolative Small-polarons conduction regime in Ce_1−x_Gd_x_O_2−x/2_, probed by the EPR spectral intensity of Gd^3+^. J. Sol. State. Chem. 177, 4104–4111. 10.1016/j.jssc.2004.07.031

[B142] Omar S. Wachsman E. D. Nino J. C. (2006). A co-doping approach towards enhanced ionic conductivity in fluorite-based electrolytes. Solid State Ionics 177, 3199–3203. 10.1016/j.ssi.2006.08.014

[B143] Omar S. Wachsman E. D. Nino J. C. (2007). Higher ionic conductive ceria-based electrolytes for solid oxide fuel cells. Appl. Phys. Lett. 91:144106 10.1063/1.2794725

[B144] Omar S. Wachsman E. D. Nino J. C. (2008). Higher conductivity Sm^3+^ and Nd^3+^ co-doped ceria-based electrolyte materials. Solid State Ionics 178, 1890–1897. 10.1016/j.ssi.2007.12.069

[B145] Ou D. R. Mori T. Ye F. Takahashi M. (2006b). Oxygen vacancy ordering in heavily rare-earth-doped ceria. Appl. Phys. Lett. 89:171911 10.1063/1.2369881

[B146] Ou D. R. Mori T. Ye F. Takahashi M. Zou J. Drennan J. (2006a). Microstructures and electrolytic properties of yttrium-doped ceria electrolytes: dopant concentration and grain size dependences. Acta Mater. 54, 3737–3746. 10.1016/j.actamat.2006.04.003

[B147] Ou D. R. Mori T. Ye F. Zou J. Auchterlonie G. Drennan J. (2008a). Oxygen-vacancy ordering in lanthanide-doped ceria: dopant-type dependence and structure model. Phys. Rev. B 77:0240108 10.1103/PhysRevB.77.024108

[B148] Ou D. R. Mori T. Ye F. Zou J. Drennan J. (2008b). Comparis on between Y-doped ceria and Ho-doped ceria: Electrical conduction and microstructures. Renew. Energ. 33, 197–200. 10.1016/j.renene.2007.05.005

[B149] Pacchioni G. (2008). Modeling doped and defective oxides in catalysis with density functional theory methods: room for improvements. J. Chem. Phys. 128:182505. 10.1063/1.2819245 18532790

[B150] Papaefthimiou V. Shishkin M. Niakolas D. K. Athanasiou M. Law Y. T. Arrigo R. (2013). On the active surface state of nickel-ceria solid oxide fuel cell anodes during methane electrooxidation. Adv. Energy Mater. 3, 762–769. 10.1002/aenm.201200727

[B151] Park S. Craciun R. Vohs J. M. Gorte R. J. (1999). Direct oxidation of hydrocarbons in a solid oxide fuel cell: I. methane oxidation. J. Electrochem. Soc. 146, 3603–3605. 10.1149/1.1392521

[B152] Peterson P. F. Bozin E. S. Proffen T. Billinge S. J. L. (2003). Improved measures of quality for the atomic pair distribution function. J. Appl. Cryst. 36, 53–64. 10.1107/S0021889802018708

[B153] Plata J. J. Márquez A. M. Sanz J. F. (2012). Improving the density functional theory+U description of CeO_2_ by including the contribution of the O 2p electrons. J. Chem. Phys. 136:041101. 10.1063/1.3678309 22299851

[B154] Purton J. A. Allan N. L. Gunn D. S. (2017). Simulations of doped CeO_2_ at finite dopant concentrations. Solid State Ionics 299, 32–37. 10.1016/j.ssi.2016.09.017

[B155] Qiu X. Bozin E. S. Juhas P. Proffen T. Billinge S. J. L. (2004). Reciprocal-space instrumental effects on the real-space neutron atomic pair distribution function. J. Appl. Crystallogr.37, 110–116. 10.1107/S0021889803026670

[B156] Reddy G. K. Thrimurthulu G. Reddy B. M. (2009). A rapid microwave-induced solution combustion synthesis of ceria-based mixed oxides for catalytic applications. Catal Surv Asia 13, 237–255. 10.1007/s10563-009-9081-9

[B157] Rezaei M. Alavi S. M. Sahebdelfar S. Yan Z.-F. (2009). Synthesis of ceria doped nanozirconia powder by a polymerized complex method. J. Porous Mater. 16, 497–505. 10.1007/s10934-008-9224-9

[B158] Riegraf M. Hoerlein M. P. Costa R. Schiller G. Friedrich K. A. (2017). Sulfur poisoning of electrochemical reformate conversion on nickel/gadolinium-doped ceria electrodes. ACS Catal. 7, 7760–7771. 10.1021/acscatal.7b02177

[B159] Sacco A. (2017). Electrochemical impedance spectroscopy: fundamentals and application in dye-sensitized solar cells. Renew. Sust. Energ. Rev. 79, 814–829. 10.1016/j.rser.2017.05.159

[B160] Sardar K. Playford H. Y. Darton R. J. Barney E. R. Hannon A. C. Tompsett D. (2010). Nanocrystalline cerium-bismuth oxides: synthesis, structural characterization, and redox properties. Chem. Mater. 22, 6191–6201. 10.1021/cm1025848

[B161] Satake J. Takayama T. Yamamura H. (2010). Oxide ion conduction and dielectric relaxation for Ce_1−x_Y_x_O_2−x/2_ system. Trans. Mater. Res. Soc. Japan 35, 659–663. 10.14723/tmrsj.35.659

[B162] Sato K. Suzuki K. Yashiro K. Kawada T. Yugami H. Hashida T. (2009). Effect of Y_2_O_3_ addition on the conductivity and elastic modulus of (CeO_2_)_1−x_(YO_1.5_)_*x*_. Solid State Ionics 180, 1220–1225. 10.1016/j.ssi.2009.06.003

[B163] Sayle D. C. Sayle T. X. T. (2007). Atomistic Models and Molecular Dynamics, in Synthesis, Properties, and Applications of Oxide Nanomaterials. Hoboken, NJ: John Wiley & Sons, Inc.

[B164] Scavini M. Chiodelli G. Spinolo G. Flor G. (1994). Electrons and Holes in undoped Nd2CuO4. Physica C 230, 412–418. 10.1016/0921-4534(94)90859-1

[B165] Scavini M. Coduri M. Allieta M. Brunelli M. Ferrero C. (2012). Probing complex disorder in Ce_1−x_Gd_x_O_2−x/2_ using the pair distribution function analysis. Chem. Mater. 24, 1338–1345. 10.1021/cm203819u

[B166] Scavini M. Coduri M. Allieta M. Masala P. Cappelli S. Oliva C. (2015). Percolating hierarchical defect structures drive phase transformation in Ce_1−x_Gd_x_O_2−x/2_: a total scattering study. IUCr J. 2, 511–522. 10.1107/S2052252515011641 PMC4547819 26306193

[B167] Scavini M. Coduri M. Allieta M. Mollica L. Brunelli M. Malavasi L. (2010). Unveiling the mesoscopic disorder induced by Al-doping in SmBa_2_Cu_3−x_Al_x_O_6+δ_ superconductors by mean of the reciprocal and real space analysis of Synchrotron Radiation XRPD data. J. Phys. Chem. C 114, 19509–19520. 10.1021/jp106805z

[B168] Sen S. Avila-Paredes H. J. Kim S. (2008). Direct spectroscopic observation of size-dependent vacancy distribution in Y-doped CeO_2_. J. Mater. Chem. 18, 3915–3917. 10.1039/b810888a

[B169] Shannon R. D. (1976). Revised effective ionic radii and systematic studies of interatomic distances in halides and chalcogenides. Acta Crystallogr. Sect. A 32, 751–767. 10.1107/S0567739476001551

[B170] Shi H. Hussain T. Ahuja R. Kang T. W. Luo W. (2016). Role of vacancies, light elements and rare-earth metals doping in CeO_2_. Sci. Rep. 6:31345. 10.1038/srep31345 27554285 PMC4995507

[B171] Shirbhate S. C. Yadav A. K. Acharya S. A. (2016). Extended x-ray absorption fine structure spectroscopy and x-ray absorption near edge spectroscopy study of aliovalent doped ceria to correlate local structural changes with oxygen vacancies clustering. Appl. Phys. Lett. 108:143501 10.1063/1.4945098

[B172] Shuk P. Greenblatt M. Croft M. (2000). Hydrothermal synthesis and properties of Ce_1−x_Eu_x_O_2−delta_ solid solutions. J. All. Compd. 303–304, 465–471. 10.1016/S0925-8388(00)00627-7

[B173] Skone J. H. Govoni M. Galli G. (2014). Self-consistent hybrid functional for condensed matter systems. Phys. Rev. B 89:195112 10.1103/PhysRevB.89.195112

[B174] Steele B. C. H. (2000). Appraisal of Ce_1−y_Gd_y_O_2−y/2_ electrolytes for IT-SOFC operation at 500°C. Solid State Ionics 129, 95–110. 10.1016/S0167-2738(99)00319-7

[B175] Sun L. Huang X. Wang L. Janotti A. (2017). Disentangling the role of small polarons and oxygen vacancies in CeO_2_. Phys. Rev. B 95:245101 10.1103/PhysRevB.95.245101

[B176] Szász J. Wankmüller F. Joos J. Wilde V. Störmer H. Gerthsen D. (2017). Correlating cathode/electrolyte interface characteristics to SOFC performance. ECS Trans. 77, 27–34. 10.1149/07710.0027ecst

[B177] Taniguchi T. Watanabe T. Sugiyama N. Subramani A. K. Wagata H. Matsushita N. (2009). Identifying defects in ceria-based nanocrystals by UV resonance raman spectroscopy. J. Phys. Chem. C 113, 19789–19793. 10.1021/jp9049457

[B178] Tran F. Blaha P. Schwarz K. (2006). How close are the slater and becke–roussel potentials in solids? J. Chem. Theory Comput. 11, 4717–4726. 10.1021/acs.jctc.5b00675 26500460 PMC4606396

[B179] Travlos A. Boukos N. Apostolopoulos G. Dimoulas A. (2003). Oxygen vacancy ordering in epitaxial layers of yttrium oxide on Si (001). Appl. Phys. Lett. 82, 4053–4055. 10.1063/1.1581985

[B180] Trovarelli A. (1996). Catalytic properties of ceria and CeO_2_-containing materials. Catal. Rev. 38, 439–520. 10.1080/01614949608006464

[B181] Trovarelli A. (2002). Catalysis by Ceria and Related Materials. London: Imperial College Press.

[B182] Trovarelli A. Llorca J. (2017). Ceria catalyst at the nanoscale: How do crystal shapes shape catalysis? ACS Catal. 7, 4716–4735. 10.1021/acscatal.7b01246

[B183] Tsunekawa S. Ishikawa K. Li Z. Kawazoe Y. Kasuya A. (2000). Origin of anomalous lattice expansion in oxide nanoparticles. Phys. Rev. Lett. 85, 3440–3443. 10.1103/PhysRevLett.85.3440 11030916

[B184] Tuller H. L. (2000). Ionic conduction in nanocrystalline materials. Solid State Ionics 131, 143–157. 10.1016/S0167-2738(00)00629-9

[B185] Tuller H. L. Nowick A. S. (1975). Doped ceria as a solid oxide electrolyte. J. Electrochem. Soc. 122, 255–259. 10.1149/1.2134190

[B186] Tuller H. L. Nowick A. S. (1977). Small polaron electron transport in reduced CeO_2_ single crystals. J. Phys. Chem. Solids 38, 859–867. 10.1016/0022-3697(77)90124-X

[B187] Tuller H. L. Nowick A. S. (1979). Defect structure and electrical properties of nonstoichiometric CeO_2_ single crystals. J. Electrochem. Soc. 126, 209–217. 10.1149/1.2129007

[B188] van de Walle A. Asta M. Ceder G. (2002). The alloy theoretical automated toolkit: a user guide. Calphad 26, 539–553. 10.1016/S0364-5916(02)80006-2

[B189] Van Herle J. Horita T. Kawada T. Sakai N. Yokokawa H. Dokiya M. (1998). Oxalate coprecipitation of doped ceria powder for tape casting. Ceram. Int. 24, 229–241. 10.1016/S0272-8842(97)00007-2

[B190] Vanpoucke D. E. Bultinck P. Cottenier S. Van Speybroeck V. Van Driessche I. (2014). Aliovalent doping of CeO_2_: DFT study of oxidation state and vacancy effects. J. Mater. Chem. A 2, 13723–13737. 10.1039/C4TA02449D

[B191] Vives S. Meunier C. (2015). Defect cluster arrangements and oxygen vacancy migration in Gd doped ceria for different interatomic potentials. Solid State Ionics 283, 137–144. 10.1016/j.ssi.2015.10.003

[B192] Wallenberg R. Withers R. Bevan D. J. M. Thompson J. G. Barlow P. Hyde B. G. (1989). The Fluorite-related “solid solutions” of CeO_2_,-Y_2_O_3_ I: A re-examination by electron microscopy and diffraction. J. Less-Common Metals 156, 1–16.

[B193] Wang D. Y. Park D. S. Griffith J. Nowick A. S. (1981). Oxygen-ion conductivity and defect interaction in Yttria-doped ceria. Solid State Ionics 2, 95–105. 10.1016/0167-2738(81)90005-9

[B194] Wang F.-Y. Chen S. Cheng S. (2004). Gd^3+^ and Sm^3+^ co-doped ceria based electrolytes for intermediate temperature solid oxide fuel cells. Electrochem. Comm. 6, 743–746. 10.1016/j.elecom.2004.05.017

[B195] Wang S. Chen S. Navrotsky A. Martin M. Kim S. Munir Z. A. (2010). Modified polyol-mediated synthesis and consolidation of Gd-doped ceria nanoparticles. Solid State Ionics 181, 372–378. 10.1016/j.ssi.2010.01.021

[B196] Wang Y. Kageyama H. Mori T. Yoshikawa H. Drennan J. (2006). Local structures around Y and Ce cations in 10 mol% Y_2_O_3_ doped ceria ceramics by EXAFS spectroscopy. Solid State Ionics 177, 1681–1685. 10.1016/j.ssi.2006.03.012

[B197] Wilkes M. F. Hayden P. Bhattacharya A. K. (2003). Catalytic studies on ceria lanthana solid solutions III. Surface segregation and solid state studies. J. Catal. 219, 305–309. 10.1016/S0021-9517(03)00046-0

[B198] Yamazaki S. Matsui T. Ohashi T. Arita Y. (2000). Defect structures in doped CeO_2_ studied by using XAFS spectrometry. Solid State Ionics 136–137, 913–920. 10.1016/S0167-2738(00)00569-5

[B199] Yamazaki S. Matsui T. Sato T. Arita Y. Nagasaki T. (2002). EXAFS study of reduced ceria doped with lanthanide oxides. Solid State Ionics 154–155, 113–118. 10.1016/S0167-2738(02)00471-X

[B200] Yashima M. Kobayashi S. Yasui T. (2006). Crystal structure and the structural disorder of ceria from 40 to 1497 C. Solid State Ionics 177, 211–215. 10.1016/j.ssi.2005.10.033

[B201] Yashima M. Takizawa T. (2010). Atomic displacement parameters of ceria doped with rare-earth oxide Ce_0.8_R_0.2_O_1.9_ (R) La, Nd, Sm, Gd, Y, and Yb) and correlation with oxide-ion conductivity. J. Phys. Chem. C 114, 2385–2392. 10.1021/jp910925t

[B202] Ye F. Mori T. Ou D. R. Zou J. Auchterlonie G. Drennan J. (2008). Compositional and structural characteristics of nano-sized domains in gadolinium-doped ceria. Solid State Ionics 179, 827 −831. 10.1016/j.ssi.2008.02.034

[B203] Ye F. Mori T. Ou D. R. Zou J. Drennan J. (2009). A structure model of nano-sized domain in Gd-doped ceria. Solid State Ionics 180, 1414–1420. 10.1016/j.ssi.2009.08.013

[B204] Yeriskin I. Nolan M. (2010). Doping of ceria surfaces with lanthanum: A DFT + U study. J. Phys. 22:135004. 10.1088/0953-8984/22/13/135004 21389507

[B205] Yoshida H. Deguchi H. Miura K. Horiuchi M. Inagaki T. (2001). Investigation of the relationship between the ionic conductivity and the local structures of singly and doubly doped ceria compounds using EXAFS measurement. Solid State Ionics 140, 191–199. 10.1016/S0167-2738(01)00854-2

[B206] Zajac W. Molenda J. (2008). Electrical conductivity of doubly doped ceria. Solid State Ionics 179, 154–158 10.1016/j.ssi.2007.12.047

[B207] Zen E. (1956). Validity of “Vegard's Law. Am. Mineral. 41, 523–524.

[B208] Žguns P. A. Ruban A. V. Skorodumova N. V. (2017). Ordering and phase separation in Gd-doped ceria: A combined DFT, cluster expansion and Monte Carlo study. Phys. Chem. Chem. Phys. 19, 26606–26620. 10.1039/C7CP04106C 28949350

[B209] Žguns P. A. Ruban A. V. Skorodumova N. V. (2018). Phase diagram and oxygen–vacancy ordering in the CeO_2_ –Gd_2_O_3_ system: a theoretical study. Phys. Chem. Chem. Phys. 20, 11805–11818. 10.1039/C8CP01029C 29658037

[B210] Zha S. Xia C. Meng G. (2003). Effect of Gd (Sm) doping on properties of ceria electrolyte for solid oxide fuel cells. J. Power Sources 115, 44–48. 10.1016/S0378-7753(02)00625-0

[B211] Zhan Z. L. Wen T.-L. Tu H. Lu Z.-Y. (2001). AC impetedence investigation of samarium-doped ceria. J. Electrochem. Soc. 148, A427–A432. 10.1149/1.1359198

[B212] Zhang J. Ke C. Wu H. Yu J. Wang J. (2016). Lattice thermal expansion and solubility limits of neodymium-doped ceria. J. Solid State Chem. 243, 57–61. 10.1016/j.jssc.2016.08.009

[B213] Zhang T. S. Hing P. Huang H. T. Kilner J. (2002). Ionic conductivity in the CeO_2_-Gd_2_O_3_ system 0.05 ≤ Gd/Ce ≤ 0.4) prepared by oxalate coprecipitation. Solid State Ionics 148, 567–573. 10.1016/S0167-2738(02)00121-2

[B214] Zhang T. S. Ma J. Kong L. B. Chan S. H. Kilner J. (2004). Aging behavior and ionic conductivity of ceria-based ceramics: a comparative study. Solid State Ionics 170, 209–217. 10.1016/j.ssi.2004.03.003

[B215] Zhu W. Z. Deevi S. C. (2003). A review on the status of anode materials for solid oxide fuel cells. Mater. Sci. Eng. A 362, 228–239. 10.1016/S0921-5093(03)00620-8

